# Clustered Protocadherins Are Required for Building Functional Neural Circuits

**DOI:** 10.3389/fnmol.2017.00114

**Published:** 2017-04-24

**Authors:** Sonoko Hasegawa, Hiroaki Kobayashi, Makiko Kumagai, Hiroshi Nishimaru, Etsuko Tarusawa, Hiro Kanda, Makoto Sanbo, Yumiko Yoshimura, Masumi Hirabayashi, Takahiro Hirabayashi, Takeshi Yagi

**Affiliations:** ^1^KOKORO-Biology Group, Laboratories for Integrated Biology, Graduate School of Frontier Biosciences, Osaka UniversitySuita, Japan; ^2^Japan Science and Technology Agency-Core Research for Evolutional Science and Technology, CREST, Osaka UniversitySuita, Osaka, Japan; ^3^System Emotional Science, Graduate School of Medicine, University of ToyamaToyama, Japan; ^4^Section of Visual Information Processing, National Institute for Physiological Sciences, National Institutes of Natural SciencesOkazaki, Japan; ^5^Section of Mammalian Transgenesis, Center for Genetic Analysis of Behavior, National Institute for Physiological SciencesOkazaki, Japan

**Keywords:** genome engineering, synapse, synchronous activity, homophilic interaction, locomotion, brainstem reticular formation, hippocampus, neuronal death

## Abstract

Neuronal identity is generated by the cell-surface expression of clustered protocadherin (Pcdh) isoforms. In mice, 58 isoforms from three gene clusters, *Pcdh*α*, Pcdh*β, and *Pcdh*γ, are differentially expressed in neurons. Since *cis*-heteromeric Pcdh oligomers on the cell surface interact homophilically with that in other neurons *in trans*, it has been thought that the Pcdh isoform repertoire determines the binding specificity of synapses. We previously described the cooperative functions of isoforms from all three *Pcdh* gene clusters in neuronal survival and synapse formation in the spinal cord. However, the neuronal loss and the following neonatal lethality prevented an analysis of the postnatal development and characteristics of the clustered-Pcdh-null (Δ*αβγ*) neural circuits. Here, we used two methods, one to generate the chimeric mice that have transplanted Δ*αβγ* neurons into mouse embryos, and the other to generate double mutant mice harboring null alleles of both the *Pcdh* gene and the proapoptotic gene *Bax* to prevent neuronal loss. First, our results showed that the surviving chimeric mice that had a high contribution of Δ*αβγ* cells exhibited paralysis and died in the postnatal period. An analysis of neuronal survival in postnatally developing brain regions of chimeric mice clarified that many Δ*αβγ* neurons in the forebrain were spared from apoptosis, unlike those in the reticular formation of the brainstem. Second, in Δ*αβγ/Bax* null double mutants, the central pattern generator (CPG) for locomotion failed to create a left-right alternating pattern even in the absence of neurodegeneraton. Third, calcium imaging of cultured hippocampal neurons showed that the network activity of Δ*αβγ* neurons tended to be more synchronized and lost the variability in the number of simultaneously active neurons observed in the control network. Lastly, a comparative analysis for *trans*-homophilic interactions of the exogenously introduced single Pcdh-γA3 isoforms between the control and the Δ*αβγ* neurons suggested that the isoform-specific *trans*-homophilic interactions require a complete match of the expressed isoform repertoire at the contacting sites between interactive neurons. These results suggested that combinations of clustered Pcdh isoforms are required for building appropriate neural circuits.

## Introduction

Neuronal identity is generated by the cell-surface expression of clustered protocadherin (Pcdh) isoforms, recognition molecules which are highly expressed in the nervous system. In mice, the clustered *Pcdh* family includes 58 members encoded by three gene clusters, *Pcdh*α*, Pcdh*β, and *Pcdh*γ, which are located on the same chromosome (Kohmura et al., 1998; Wu and Maniatis, 1999). Individual neurons constitutively express 5 *Pcdh-C*-type isoforms (α*C1*, α*C2*, γ*C3*, γ*C4*, and γ*C5*) and stochastically express distinct combinations of the remaining 53 isoforms of *Pcdh*α*, Pcdh*β, and *Pcdh*γ (Esumi et al., 2005; Kaneko et al., 2006; Hirano et al., 2012). Unlike classical cadherins, clustered Pcdh isoforms form distinct combinations of *cis*-heteromeric oligomers that undergo *trans*-homophilic interactions on the cell surface (Murata et al., 2004; Schreiner and Weiner, 2010; Thu et al., 2014; Nicoludis et al., 2015; Rubinstein et al., 2015). A cell-aggregation assay with K562 cells expressing exogenous Pcdh isoforms demonstrated that the homophilic interactions depend on specific combinations of multiple Pcdh isoforms between different cells. Even a single mismatched Pcdh isoform can interfere with these homophilic interactions (Schreiner and Weiner, 2010; Thu et al., 2014). Given the expression of Pcdh isoforms at the contacting sites between neurons during neural development, the Pcdh isoform repertoire in each neuron may determine the binding specificity between neurons.

Analyses of *Pcdh* knockout mice have shown that the clustered Pcdh isoforms are required not only for synapse formation but for multiple aspects of recognition events, such as axonal projection, dendritic self-avoidance, and dendritic arbor complexity (Hasegawa et al., 2008; Katori et al., 2009; Prasad and Weiner, 2011; Garrett et al., 2012; Lefebvre et al., 2012; Suo et al., 2012; Kostadinov and Sanes, 2015; Molumby et al., 2016). Among the three types of *Pcdh*-mutant mice, *Pcdh*γ null mutants exhibit the most severe phonotype: they die after birth with repetitive tremors associated with massive interneuron death and synapse loss in the spinal cord (Wang et al., 2002; Hambsch et al., 2005; Weiner et al., 2005; Chen et al., 2012; Hasegawa et al., 2016). Moreover, similar phenotypes have also been observed in γ*C* TKO mutants, which lack only the γ*C3*, γ*C4*, and γ*C5* genes (Chen et al., 2012). Genetically blocking apoptosis with *Bax* mutants still could not rescue the synapse loss phenotype of *Pcdh*γ null mutants, confirming the hypothesis that the Pcdhγ isoforms regulates the synapse formation (Weiner et al., 2005).

In our previous study, however, we showed that not only Pcdhγ, but also Pcdhα or Pcdhβ isoforms play a role in neuronal survival and synapse formation, by analyzing Δ*αβγ* mice, which lack all 58 isoforms in all three *Pcdh* clusters. Deleting the *Pcdh*α or *Pcdh*β cluster also causes a mild neuronal death phenotype, and the phenotypic severity of neuronal death and synaptic loss increases with the number of deleted clusters (Δγ<Δ*βγ* <Δ*αβγ*), suggesting that all three *Pcdh* clusters cooperatively contribute to neuronal survival and functional neural circuit formation (Hasegawa et al., 2016). Tarusawa et al. analyzed the connectivity of Δ*αβγ* cortical neurons lacking all three clustered *Pcdh* genes transplanted in wild-type (WT) mice to analyze the postnatal brain, and showed that the Pcdh isoforms regulate the neural network topology (Tarusawa et al., 2016).

However, several questions remain to be studied; firstly, neuronal survival during postnatal development in the midbrain or forebrain area of Δ*αβγ* mouse has not been studied due to the lethality of pups; secondly, the functionality or activity propagation characteristics of the neural circuits in the Δ*αβγ* mouse have not been studied.

In this study, firstly, to overcome the neonatal lethality of Δ*αβγ* mice and the inability to analyze postnatal mice, we developed chimeric mice that have Δ*αβγ* neurons derived from induced pluripotent stem cells (iPSCs) from Δ*αβγ* embryonic fibroblasts. The resultant chimeric mice survived the postnatal developing period, and we found a lower rate of apoptosis in the neurons of the olfactory bulb, cortex, hippocampus, and cerebellum, compared to the reticular formation of the brainstem and spinal cord, that showed massive apoptosis.

Secondly, for the first time, we analyzed the network characteristics of the Δ*αβγ* neural circuits lacking all 58 Pcdh isoforms. We analyzed two neural circuits; the central pattern generator (CPG) in the spinal cord, and the *in vitro* network of cultured dissociated hippocampal neurons. To analyze the CPG, we prevented the neuronal death in the spinal cord of Δ*αβγ* mice by deleting the *Bax* gene. We found that the Δ*αβγ:Bax*^−/−^ CPG were non-functional and unable to create a left-right alternating pattern even in the absence of apoptotic interneuron loss. To analyze the *in vitro* cultured network of hippocampal neurons, we performed multi-neuron calcium imaging. We found that the network activity of Δ*αβγ* hippocampal neurons differed from control neurons in that the activity tended to be more synchronized and failed to generate the variability in the number of simultaneously active neurons.

Intrinsically, Δ*αβγ* neurons, which lack all 58 Pcdh isoforms of the *Pcdh* gene clusters, are ideal neurons to study the recognition specificity of the Pcdh isoforms at the synapse. Lastly, for the first time, we visualized *trans*-homophilic interactions mediated by the exogenously introduced fluorescently-tagged Pcdh isoforms in living neurons. We confirmed that the isoform-specific interactions between overexpressed γA3 proteins in two contacting Δ*αβγ* neurons were observed more frequently than that in control neurons in which the Pcdh isoform repertoire is thought to differ at each synapse.

Conclusively, all these results indicated that diverse combinations of the isoforms generated by *Pcdh*α*, Pcdh*β, and *Pcdh*γ gene clusters are required for building appropriate neural circuits.

## Materials and methods

### Animal experiments

All of the experimental procedures were conducted according to the Guide for the Care and Use of Laboratory Animals of the Science Council of Japan and were approved by the Animal Experiment Committee of Osaka University.

### Generation of *Δ*αβγ** mice

Δ*αβγ* mice were generated as described previously (Hasegawa et al., 2016). Our initially produced *Pcdhabg*^*del*/*del*^ mutants contained an additional deletion of the *TAF7* gene, which is located between the *Pcdh*β and *Pcdh*γ clusters, and *TAF7*-deficient mice are known to be early embryonic lethal (Gegonne et al., 2012). Therefore, to rescue the *TAF7* gene, we produced a *TG*^*taf*7^ transgenic mouse line using a BAC plasmid of a 20-kb region between the *Pcdh*β and *Pcdh*γ gene clusters that included the *TAF7* gene. By crossing the *TG*^*taf*7^ transgenic mice and heterozygous *Pcdhabg*^*del*/+^ mutants, we obtained homozygous *Pcdhabg*^*del*/*del*^ mutants with the *TG*^*taf*7^ transgene, Δ*αβγ* mice. The *TG*^*taf*7^ transgene completely rescued the early embryonic lethality caused by the TAF7 deficiency. In the *TAF7* transgenic animals, the expression level of *TAF7* was significantly increased to more than three times of the level in the WT animal (Hasegawa et al., 2016). Then, we performed all experiments by using the *Pcdhabg*^+/+^*;TG*^*taf*7^ (+*/*+*:TG*^*taf*7^) or the *Pcdhabg*^*del*/+^*;TG*^*taf*7^ mice (control).

### Generation of iPSCs and chimeric mice

The WT and Δ*αβγ*-iPSCs chimeric mice were generated as described previously (Tarusawa et al., 2016). The *TAF7* transgene is included in the Δ*αβγ*-iPSC chimeras, but not in the WT-iPSC chimeras.

### Construction of expression vectors

The *Pcdh*-γ*A3* (γ*A3*) and -γ*B2* (γ*B2*) coding sequences were amplified from mouse whole-brain cDNA by RT-PCR and were subcloned into pBluescript, and the Venus or tdTomato Open Reading Frame was inserted in-frame into the Pcdh C-terminal. The Pcdh-Venus and Pcdh-tdTomato DNA fragments were ligated into the pCX plasmid. All expression vector constructs were confirmed by DNA sequencing.

### Immunohistochemistry

Immunohistochemistry was performed as described previously (Hasegawa et al., 2008) using the following antibodies: anti-NeuN (Chemicon), anti-ChAT (Chemicon), anti-Chx10 (Santa Cruz), anti-DsRed (Clontech), anti-FoxP2 (Sigma), anti-GFP (Invitrogen), anti-GFP (Nacalai), anti-gephyrin (Synaptic Systems), anti-GFAP (Sigma), anti-PSD95 (Millipore), anti-somatostatin (ImmunoStar), anti-VGAT (Frontier Institute), anti-cleaved-caspase-3 (Cell Signaling Technology), anti-pan-axonal-neurofilament (SMI312, Covance), anti-synapsin I (Calbiochem), and Alexa Fluor 488–conjugated α-bungarotoxin (Molecular Probes).

### Histology

The diaphragm was removed, fixed for 2 h in 4% paraformaldehyde in 0.1 M phosphate buffer (pH 7.3), and rinsed with 0.1M glycine in phosphate-buffered saline (PBS). Next, the muscles were dissected, incubated in PBS containing 10 mg/ml bovine serum albumin and Alexa 488–conjugated anti-α-bungarotoxin antibody (Molecular Probes) overnight at 4°C, washed in PBS, and mounted with anti-fading reagent (Dianova). When we conducted the antibody staining of the brain and spinal cord sections, the mice were fixed in 4% paraformaldehyde in 0.1M phosphate buffer by transcardial perfusion and postfixed for 2 h at 4°C. Following cryoprotection in 25% sucrose, the embryos were embedded in OCT compound (Miles), and 20 μm sections were cut on a cryostat (Leica CM3050, Germany).

### Primary culture and immunocytochemistry

The hippocampus and brainstem were isolated from E16.5 and E12.5–E14.5 embryos, respectively, and digested with papain (Worthington Biochemical Corporation). The dissociated cells were nucleofected with the γA3-Venus (or -tdTomato) or γB2-tdTomato plasmid using the P3 Primary Cell Electroporation Kit (Lonza, Germany). The cells were resuspended, plated on 1 mg/ml poly-L-lysine–coated glass coverslips, and cultured in Minimum Essential Medium (Invitrogen) containing 5% fetal bovine serum, B27 supplement (Invitrogen), and GlutaMax I (Invitrogen). After 48 h, the medium was replaced with Neurobasal serum-free medium (Invitrogen) supplemented with B27 and GlutaMax I. Neurons were fixed with 4% paraformaldehyde or methanol for 15 min and processed for immunocytochemistry.

### Electrophysiology

Spinal cords from E18.5–P0 mice were removed and analyzed as described previously (Hasegawa et al., 2016). Data were presented as the mean ± SEM. Statistical comparisons were performed by Student's *t*-test.

### Calcium imaging

Hippocampal cells were dissociated from E16.5 mouse embryos by brief trypsinization (0.25% trypsin for 10 min at 37°C) and trituration through a fire-polished Pasteur pipette, and were plated onto a 9.5-mm multi-well glass-bottom dish (D141400, Matsunami, Japan) at 550–600 cells/mm^2^. On DIV 20–21, the culture medium was replaced with a calcium-indicator loading solution consisting of 0.0005% Oregon Green BAPTA-1AM, 0.01% Pluronic F-127, and 0.005% Cremophor EL in aCSF (127 mM NaCl, 26 mM NaHCO_3_, 1.5 mM KCl, 1.24 mM KH_2_PO_4_, 1.4 mM MgSO_4_, 2.4 mM CaCl_2_, and 10 mM glucose). The cells were incubated for 1 h in a 37°C humidified incubator and then washed several times with aCSF to remove the loading solution. Live calcium imaging was performed with an LSM780 confocal laser scanning microscope equipped with the Incubation System XL (Zeiss, Germany). A 105.9 μm × 105.9 μm visual field was imaged for 2 min with an imaging interval of 100 ms. Data derived from the *Pcdh*α*βγ*^+/+^*:TG*^*taf*7^ and *Pcdh*α*βγ* ^*del*/+^*:TG*^*taf*7^ genotypes were used as controls.

### Imaging

Fluorescent images were captured on an FV1000 confocal microscope (Olympus, Japan), an LSM780 confocal microscope (Zeiss, Germany), a BZ9000 microscope (Keyence, Japan), or a BX-X710 microscope (Keyence, Japan). Images were prepared for printing with Adobe Photoshop Elements Editor. Synaptic density was quantified using images obtained on an Olympus confocal microscope with an X2 digital zoom and X60 oil immersion objective lens. The examiners of the images used for comparisons were blinded to the conditions. Data were quantified with the attached software (Keyence) or Image J64 software (NIH).

### Statistical analysis

Statistical analyses were conducted with Prism ver. 5 (GraphPad Software, Inc.) using Student's *t*-test, one-way analysis of variance (ANOVA), and *post-hoc* Tukey tests. The Mann-Whitney *U*-test was applied for the calcium imaging. The values shown in all of the graphs were expressed as the mean ±SEM.

## Results

### Mosaic analysis using chimeric mice with *Δ*αβγ** iPSCs

In this study, we aimed to examine the phenotype of the Δ*αβγ* mice which lack all 58 clustered Pcdh isoforms, in the postnatally developing brain area that was not studied in our previous report due to the neonatal lethality of the mutant mice (Hasegawa et al., 2016). For this purpose, we generated chimeric mice that have transplanted Δ*αβγ* neurons integrated in the WT neural network.

Before discussing the survival of transplanted Δ*αβγ* neurons, we first described the neuronal death in Δ*αβγ* mice in more detail. Since our previous report mainly studied the spinal cord, in this study we focused on neuronal death in the brainstem. First, we studied when the neuronal death in the brainstem appeared in the Δ*αβγ* mice. Interestingly, at E15.5, the neurofilament or the cleaved caspase-3 staining patterns were indistinguishable between the two genotypes (Supplementary Figures 1A–B'). However, in E16.5 Δ*αβγ* mice, many apoptotic signals were present along axons within the medulla, but not in the control mice (Supplementary Figures 1C–C'). At E18.5, the medulla of Δ*αβγ* mice was undersized, and the net-like pattern was disrupted (Supplementary Figures 1G–G'). NeuN staining revealed neuronal loss, while the facial nuclei appeared normal (Supplementary Figures 1H–H'). Thus, Δ*αβγ* neurons differentiated normally and extended neurites until E15.5, but underwent extensive neuronal death throughout the brainstem from E16.5 to E18.5. These results suggest that the neuronal death we observed occurred after the neurons started to make synaptic contacts.

Moreover, the respiratory rhythm-generating center, the pre-Bötzinger complex (preBötC) of the Δ*αβγ* mice appeared normal until E16.5 (Supplementary Figures 1D–D'), but the preBötC somatostatin^+^ populations had almost disappeared by E18.5 (Supplementary Figures 1E–E',F–F'). Consistent with this, the lungs of newborn Δ*αβγ* mice have abnormally small alveolar spaces, and the pups die of respiratory failure shortly after birth (Hasegawa et al., 2016, Supplementary Movie 1). We also noted that the time course of the death of somatostatin^+^ respiratory neurons was consistent with that of the generation of respiratory rhythmic activity within the preBötC (Pagliardini et al., 2008).

**Figure 1:**
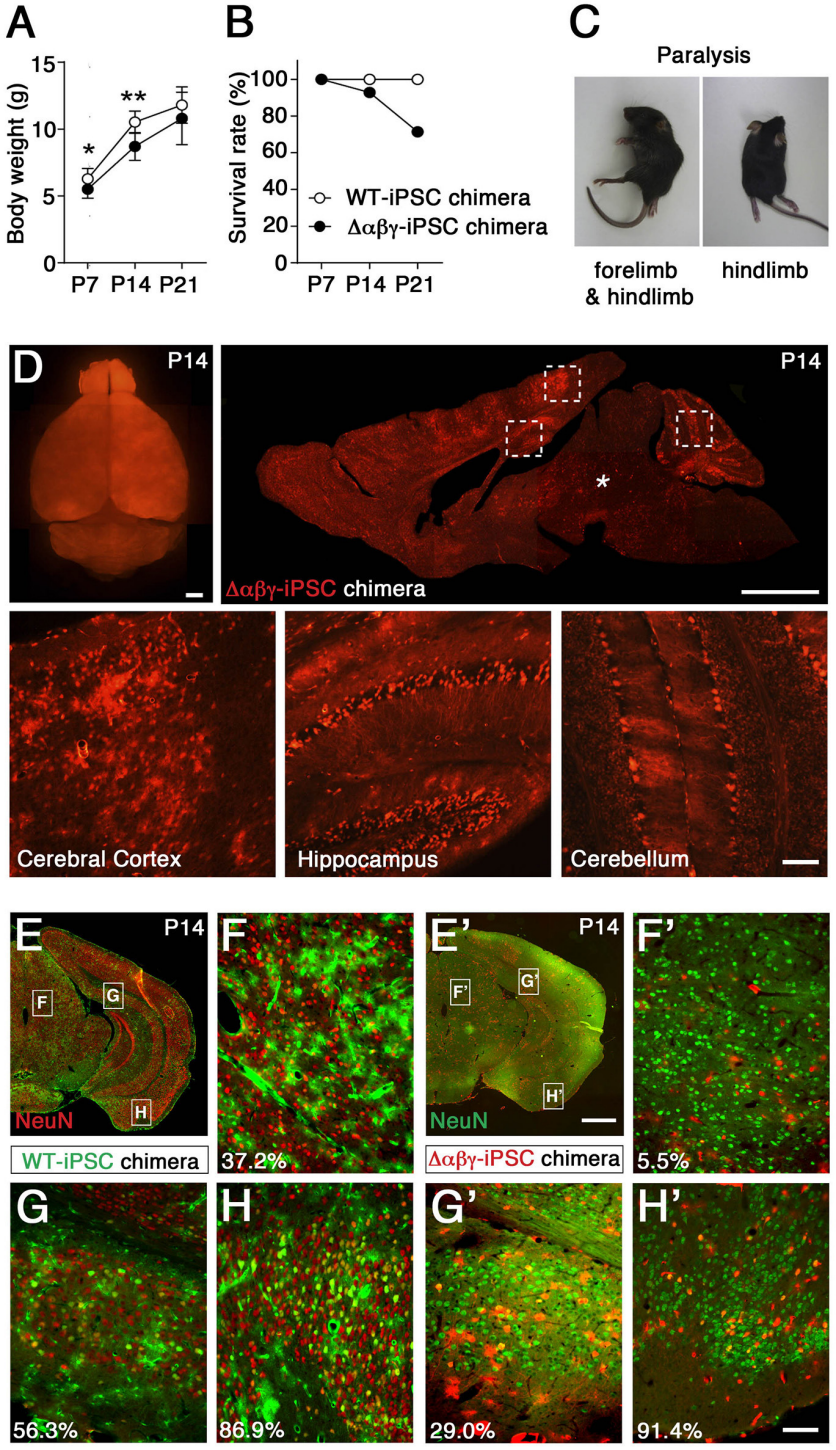
**Generation and analysis of chimeric mice with Δ***αβγ***-iPSCs. (A)** Body weights were lower in Δ*αβγ*-iPSC chimeric mice (*n* = 14 P7, 13 P14, and 10 P21 mice) than in WT-iPSC chimeric mice (*n* = 5 P7, 5 P14, and 5 P21 mice). Data are shown as mean ± *SD*. ^*^P < 0.05, ^**^P < 0.01 for WT vs. Δ*αβγ* by Student's *t*-test. **(B)** Survival of chimeric mice (WT-iPSC *n* = 20; Δ*αβγ*-iPSC *n* = 14). **(C)** Paralysis was noted in some Δ*αβγ*-iPSC chimeric mice at about P14. **(D)** A P14 chimeric mouse showing intense tdTomato^+^ signals in several regions of the brain, including the cerebral cortex, hippocampus, and cerebellum (outlined in squares), but not the brainstem (^*^). **(E–H')** Representative images of WT-iPSC **(E–H)** or Δ*αβγ*-iPSC **(E'–H')** chimeric mice. Numbers at lower left indicate the percentage of surviving neurons (yellow-colored) in each panel. Note that WT iPSCs were labeled with not tdTomato but GFP **(E–H)**. Bars: 1 mm in (**D**, upper, **E'**), 100 μm in (**D**, lower, **H'**). The TAF7 transgene was included in the Δ*αβγ*-iPSC chimeras, but not in the WT-iPSC chimeras.

Subsequently, to examine the neuronal survival of Δ*αβγ* neurons in the postnatally developing brain area, we developed chimeric mice that have Δ*αβγ* neurons derived from iPSCs from Δ*αβγ* embryonic fibroblasts, marked them with a red fluorescent protein (tdTomato), and injected them into WT blastocysts to produce chimeric mice with a red fluorescent signal (Supplementary Figure 2A; Tarusawa et al., 2016). As expected, the chimeric mice survived, but some of them that had a high number of Δ*αβγ* cells were small and died with paralysis 7 days after birth (Figures 1A–C, Supplementary Movie 2). As it was not clear whether the neuronal survival of the Δ*αβγ* neurons was dependent on the surrounding environment, we first examined whether Δ*αβγ* -iPSC neurons of the brainstem integrated into the WT neural network could survive or not. We found that the majority of red-colored Δ*αβγ* -iPSC neurons in the brainstem of Δ*αβγ* -iPSC chimeras were lost, as described above, in the Δ*αβγ* mice (Figure 1D, asterisk). These observations suggested that neuronal loss and disruption throughout the reticular formation of the brainstem, which contains ascending sensory nerves, led to paralysis even in postnatal chimeric animals.

To analyze more closely the survival rate of transplanted Δ*αβγ*-iPSC neurons in the reticular formation including midbrain, pons and medulla, we calculated the rates of differentiated neurons derived from each type of iPSC, the index which was not influenced by the large variability in the distribution rate of the transplanted neurons (chimeric rates). Three chimeric mice generated with WT or Δ*αβγ* iPSCs were analyzed. In contrast with the WT-iPSC chimera, the iPSC-derived neurons marked with green fluorescent protein (GFP), in the Δ*αβγ*-iPSC chimeras, there were few iPSC-derived neurons (<6.0%) in the hypothalamus, midbrain, pons, and medulla (Table 1). For instance, in Figure 1F, 37.2% of the GFP^+^ cells expressed NeuN (red) in the WT-iPSC chimera (GFP^+^NeuN^+^; 29 neurons, GFP^+^; 78 cells). In contrast, in Figure 1F', only 5.5% of the tdTomato^+^ cells expressed NeuN (green) in the Δ*αβγ*-iPSC chimera (tdTomato^+^NeuN^+^; 1 neuron, tdTomato^+^; 18 cells). Although a high number of Δ*αβγ*-iPSC neurons were found at E15.5 when they were still alive and appeared healthy (Supplementary Figure 2B), their number had dropped at P14. While many tdTomato^+^ signals were also found in blood vessels as well as glial cells, there were no iPSC-derived neurons (Supplementary Figure 2C). Additionally, in the Δ*αβγ*-iPSC chimeras, increased astrogliosis associated with neurodegeneration and some tdTomato^+^GFAP^+^ signals were also detected (Supplementary Figures 2C–E). Thus, Δ*αβγ* neurons differentiated normally, but died during brain development. These results suggested that Δ*αβγ* neurons in the reticular formation do not survive even when intermingled with WT neurons, at least at this rate of chimerism.

**Table 1:** **Survival ratio of chimeric populations (GFP^**+**^ in the WT-iPSC chimera and tdTomato^**+**^ in the Δ***αβγ***-iPSC chimera) and neuronal populations (NeuN^**+**^) in the hypothalamus, midbrain, pons, and medulla of the WT-iPSC and Δ***αβγ*** -iPSC chimeric mice**.

**WT-iPSC chimera (P14)**				
		**GFP^+^NeuN^+^**	**GFP^+^**	**(GFP^+^NeuN^+^)/GFP^+^ (%)**
Hypothalamus		80	126	65.1
Midbrain	PPT	208	329	40.7
	p1Rt	483	623	30.8
	mRt	427	749	43.3
Pons	PnC	952	1,132	17.8
Medulla	Gi	1,027	1,211	14.4
	IRt	585	812	33.2
Δ*αβγ***-iPSC chimera (P14)**				
		**tdTomato^+^NeuN^+^**	**tdTomato^+^**	**(tdTomato^+^NeuN^+^)/tdTomato^+^ (%)**
Hypothalamus		31	831	3.7
Midbrain	PPT	4	434	1.7
	p1Rt	1	267	0.2
	mRt	2	294	0.4
Pons	PnC	7	562	1.5
Medulla	Gi	31	464	6.0
	IRt	20	758	1.1

*In WT-iPSC chimeric animals, the (GFP^+^NeuN^+^)/GFP^+^ ratio was almost invariable among these regions. In Δαβγ-iPSC chimeric animals, the (tdTomato^+^NeuN^+^)/tdTomato^+^ ratio was notably low in the midbrain, including the pons and medulla. Data were pooled from three chimeric animals for each genotype*.

In contrast to the reticular formation, many neuronal populations expressing clustered Pcdh isoforms, such as sensory, motor, olfactory, cortical, hippocampal, and cerebellar neurons, were spared from neuronal death (Figures 1D,G',H'), in consistent with prior observations on *Pcdh*γ and *Pcdh*α knockout mice (Wang et al., 2002; Garrett et al., 2012; Hasegawa et al., 2016). Collectively, in WT-iPSC chimeras, iPSC-derived neurons had a distribution of 34.7–61.2% in all of the observed areas of the brain (Figures 1E–H; Table 2). On the other hand, in the Δ*αβγ*-iPSC chimeras, we found high numbers of iPSC-derived neurons in the olfactory bulb (75.7%) and cerebral cortex (73.7%), but fewer in the midbrain (11.2%). These results suggested that the survival of Δ*αβγ* neurons depends on the brain area or neuronal types.

**Table 2:** **Survival ratio of chimeric populations (GFP^**+**^ in the WT-iPSC chimera and tdTomato^**+**^ in the Δ***αβγ*** -iPSC chimera) and neuronal populations (NeuN^**+**^) in the olfactory bulb, cerebral cortex, and midbrain of the WT-iPSC and Δ***αβγ*** -iPSC chimeric mice**.

**WT-iPSC chimera (P14)**			
	**GFP^+^NeuN^+^**	**GFP^+^**	**(GFP^+^NeuN^+^)/GFP^+^ (%)**
Olfactory bulb	7,251	16,923	46.1
Celebral cortex	9,176	15,227	61.2
Midbrain	3,809	10,827	34.7
Δ*αβγ***-iPSC chimera (P14)**			
	**tdTomato^+^NeuN^+^**	**tdTomato^+^**	**(tdTomato^+^NeuN^+^)/tdTomato^+^ (%)**
Olfactory bulb	4,645	6,182	75.7
Celebral cortex	7,885	10,646	73.7
Midbrain	922	8,019	11.2

*In WT-iPSC chimeric animals, the (GFP^+^NeuN^+^)/GFP^+^ ratio was almost invariable among these regions. In Δαβγ-iPSC chimeric animals, the (tdTomato^+^NeuN^+^)/tdTomato^+^ ratio was notably low only in the midbrain, including the pons and medulla. Data were pooled from three animals for each genotype*.

### No rescue of the neonatal lethality, synaptic defects, or physiological locomotor-circuit malformation observed in *Δ*αβγ** mice by genetically blocking apoptosis

We subsequently analyzed the network characteristics of the Δ*αβγ* neural circuits lacking all 58 Pcdh isoforms. Since the malfunction of spinal neural circuits was potentially due to the massive neuronal death in the spinal cord and brainstem (Hasegawa et al., 2016), we tried to prevent the neuronal apoptosis by crossing Δ*αβγ* mice with mice harboring a null allele of the proapoptotic gene *Bax* (Knudson et al., 1995). The Δ*αβγ:Bax*^−/−^ double mutants died soon after birth as previous reported for *Pcdhg*^*del*/*del*^*:Bax*^−/−^ double mutants (Weiner et al., 2005). The pups showed poor reflex action and little voluntary movement (Supplementary Movie 3). The neurological defects in the Δ*αβγ:Bax*^−/−^ double mutants were similar to, but less severe than those seen in Δ*αβγ* mice. No apoptotic signals were found in the Δ*αβγ:Bax*^−/−^ double mutants and the massive death of spinal interneurons were also completely restored by the *Bax* gene deletion (Supplementary Figure 3).

**Figure 2:**
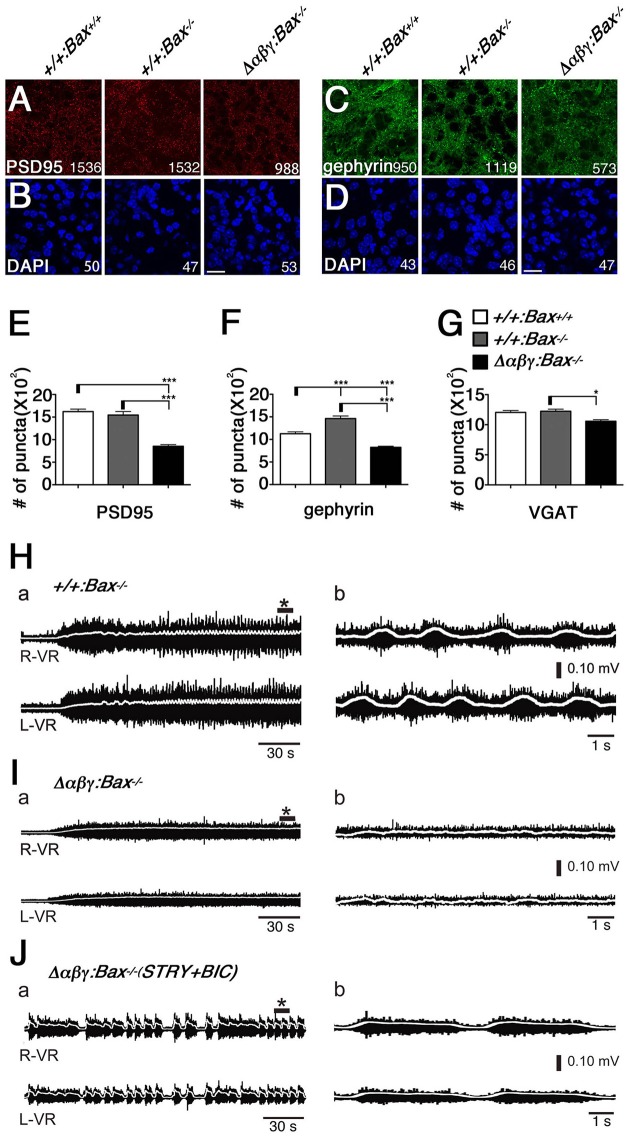
**The Δ***αβγ:Bax***^**−/−**^ double mutants showed low synaptic density and no left-right alternation of locomotor-like activity. (A–D)** The densities of the PSD95^+^ excitatory and gephyrin^+^ inhibitory synapses were reduced in the Δ*αβγ;Bax*^−/−^ double mutants. The number of synaptic puncta or cells per microscope field is shown. **(E–G)** The numbers of PSD95^+^, gephyrin^+^, and VGAT^+^ synaptic puncta were significantly decreased in the double mutants. *N* = 33, 23, 68 microscope fields for PSD95^+^ and 32, 50, 77 for gephyrin^+^. Error bars represent SEM., ^***^P < 0.001, ^*^P < 0.05 by one-way ANOVA and *post-hoc* Tukey tests. Bars: 20 μm. **(H)** Normal left-right alternating locomotor-like motor activity was recorded after NMDA and 5-HT were applied to isolated E18.5 control spinal cords, and VR activity was recorded at the right (R-VR) and left (L-VR) sides of the second (L2) lumbar segment. **(I)** The double-mutant mice did not exhibit the left-right alternation. **(J)** Left-right synchronous rhythm was evoked in the Δ*αβγ:Bax*^−/−^ double-mutant spinal cord when the inhibitory synaptic transmission was blocked by bath application of the glycinergic antagonist strychnine (STRY) and the GABAergic antagonist bicuculline (BIC). Panels in (b) show magnified views of ^*^ shown in (a). In this figure, *TG*^*taf*7^ was included in each of genotypes.

Firstly, the gross phenotype of the Δ*αβγ:Bax*^−/−^ double mutants led us to examine the synaptic defects by staining the intermediate gray in the lumbar spinal cord of the double mutants with anti-PSD95 and anti-gephyrin antibodies. Both glutamatergic and inhibitory postsynaptic synapses were present in the Δ*αβγ:Bax*^−/−^ double mutants, but at a lower density than in WT or *Bax*^−/−^ control mice (Figures 2A–D); the density of these synapses in the double mutants was approximately half that seen in *Bax*^−/−^ mice (Figures 2E,F). Thus, the postsynaptic densities were defective despite the complete rescue of the number and distribution patterns of these interneuron populations. The number of puncta positive for vesicular GABA transporter (VGAT) was decreased by ~10% in the double mutants, compared to the WT or *Bax*^−/−^ mice (Figure 2G). These results indicated that the clustered Pcdh isoforms are required to some degree to control synaptogenesis.

To assess the functional neuronal circuits in the double mutants, we then examined the CPG for locomotion in the spinal cord, which controls limb movements during walking. In the rodent, the ventral lumbar cord can generate left-right alternations (Bracci et al., 1996; Kjaerulff and Kiehn, 1996), and it is thought that the spinal CPG circuits consist of reciprocal excitatory and inhibitory connections, and that complicated interactions of various types of spinal interneurons are involved.

**Figure 3:**
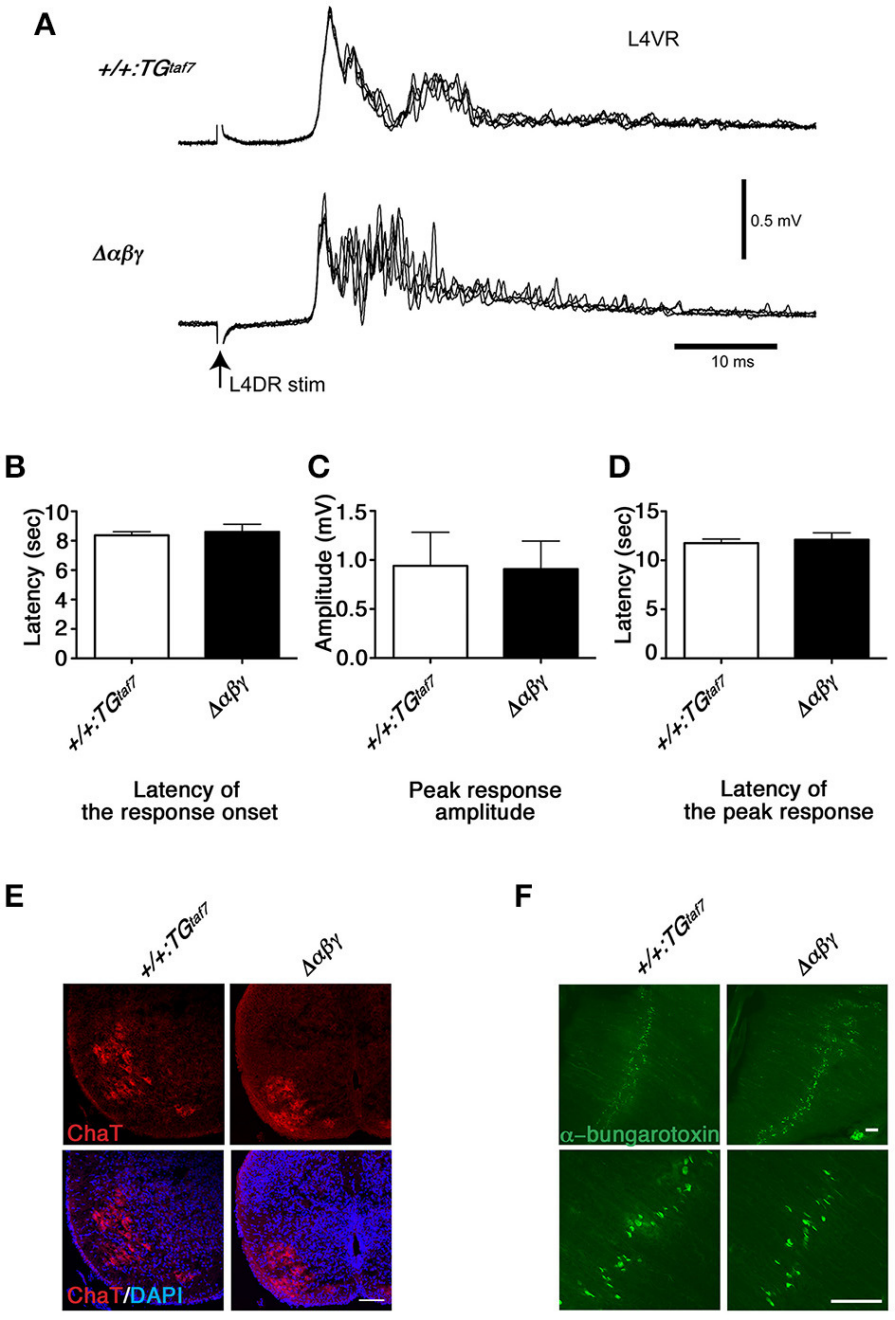
**Normal monosynaptic but abnormal polysynaptic stretch-reflex circuits. (A)** Spinal reflex at E18.5. Electric stimuli to L4DR normally activate Ia afferents and elicit an action potential in motor neurons. Monosynaptic reflex circuits appeared normal, but the polysynaptic reflex circuits were disrupted in the Δ*αβγ* mutant. No differences were detected in the latency of the response onset **(B)**, the peak response amplitude **(C)**, or the latency of the peak response **(D)**. Error bars represent SEM; data were compared by Student's *t*-test. **(E)** ChAT staining of the E18.5 Δ*αβγ* spinal cord. The number of neurons and the size and distribution of motor neurons were indistinguishable between genotypes. **(F)** Whole-mounted E18.5 diaphragm muscles were stained with FITC-conjugated α-bungarotoxin, which binds specifically to acetylcholine receptors (AChRs). As in the control (+*/*+*:TG*^*taf*7^) mice, AChR clusters were aligned normally in the central region of the muscles of the mutant mice. Bars: 100μm.

We isolated E18.5 spinal cords, induced locomotor-like rhythmic activity by bath application of N-methyl-D-aspartic acid (NMDA) and serotonin (5-HT), and recorded the electrical activity of motor neurons on the left- and right-side from the ventral roots (VRs) in the second lumbar (L2) segment. Rhythmic activity with an alternating pattern between the left and right VRs was observed in control mice (Figure 2H), but not in the Δ*αβγ:Bax*^−/−^ double mutants (Figure 2I). This phenotype is similar to that of previously studied Δ*αβγ* mice in which the neuronal loss was observed (Hasegawa et al., 2016). It is well-known that the left-right rhythm in WT mice can be synchronized by blocking the inhibitory synaptic transmission with the glycinergic antagonist strychnine (STRY) and the GABAergic antagonist bicuculline (BIC; Talpalar et al., 2011). Interestingly, even in the double mutants, the bath application of STRY and BIC evoked a left-right synchronous rhythm (Figure 2J), indicating that the excitatory connections between the left and right lumbar networks were formed normally in the double mutants. These results suggested that motor neurons in the double-mutant mice fire normally, but that their interneuronal CPG circuits cannot generate a normal locomotor pattern. In E18.5 Δ*αβγ* mice, which had massive neuronal apoptosis, the first firing of motor neurons by L4 dorsal-root (DR) stimulation was normal, but later firing patterns were different from that seen in the +*/*+*:TG*^*taf*7^ mice (Figures 3A–D). The motor neurons in Δ*αβγ* mice at E18.5 appeared normal, as assessed by staining for ChAT (a motor neuron marker) and α-bungarotoxin (a neuromuscular junction marker; Figures 3E,F). The locomotor circuits in the mouse lumbar spinal cord are organized in the late embryonic stages; in particular, the inhibitory connection underlying the left-right alternating pattern is formed at E15.5–E18.5 by bilateral interactions between networks in the left and right side of the lumbar cord (Branchereau et al., 2000; Nishimaru and Kudo, 2000). Thus, our data indicated that the clustered Pcdh isoforms are required for building neuronal circuits in the locomotor CPG during development.

### Abnormal synchronized activity in cultured *Δ*αβγ** hippocampal neurons

**Figure 4:**
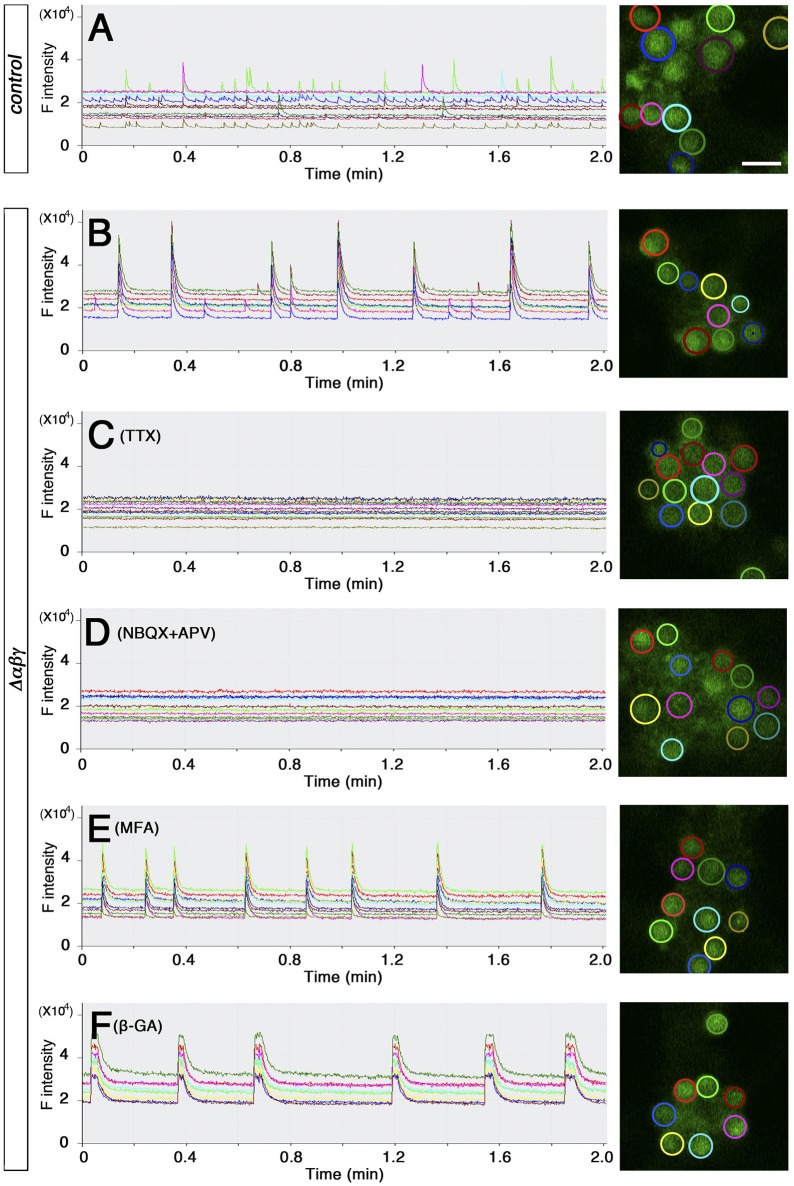
**Spontaneous activity in cultured hippocampal neurons (A,B)** calcium response from DIV 20–21 cultured hippocampal neurons loaded with OGB-1AM. Neurons in the right panel are circled in colors that match the corresponding graph at left. **(C–F)** Effects of blockers on the synchronized rhythmic calcium response of Δ*αβγ* hippocampal neurons. Calcium responses were recorded at DIV23 **(C)** or DIV20 **(D–F)**. DIV23 cultures were recorded in regular aCSF, while the earlier DIV20 cultures were recorded in modified aCSF containing 3.3 mM KCl. **(I–L)** Examples of the calcium response after the application of 100 nM TTX **(C)**, 20 μM NBQX, and 50 μM APV **(D)**, 25 μM MFA (meclofenamic acid) **(E)**, or 25 μM β-GA (18β-Glycyrrhetinic acid) **(F)**. All cultures were confirmed to exhibit a synchronized rhythmic calcium response before blocker application. Data derived from the *Pcdh*α*βγ*^+/+^*:TG*^*taf*7^ and *Pcdh*α*βγ* ^*del*/+^*:TG*^*taf*7^ genotypes were used as controls. Bar: 20μm.

The network failure observed in the Δ*αβγ:Bax*^−/−^ double mutants suggested that aside from the neuronal death phenotype, the abnormal wiring caused neural circuits in the double mutants to malfunction (Figure 2). To clarify the defects in the organization of the Δ*αβγ* networks, we visualized the calcium dynamics by calcium imaging in dissociated hippocampal neurons. The neurons aggregated in culture, forming clusters of 10–20 (or more) neurons that were interconnected by axons. We imaged and compared the calcium dynamics in neighboring neurons within a cluster at an imaging interval of 100 ms (Figures 4A,B). Spontaneous transient changes in [Ca^2+^]i were observed as early as DIV 12–14, and changes in [Ca^2+^]i synchronicity developed by the end of the third week in culture. The emergence of synchronized activity has been reported in both slice preparations and cultures of dissociated hippocampal neurons (Crépel et al., 2007; Schroeter et al., 2015; Pu et al., 2013). As shown in Figure 4A, control neurons had distinct temporal calcium-response profiles, with an occasional synchronized response (Supplementary Movie 4; compare the red and light green responses in Figure 4A). Consistent with the histological and electrophysiological results already mentioned, the Δ*αβγ* networks were as active as the control networks, indicating that the Δ*αβγ* neurons could form functional synapses in the absence of clustered Pcdh isoforms. Interestingly, the Δ*αβγ* neuronal responses tended to be more synchronized than the control neuronal responses (Supplementary Movie 5; Figure 4B). The calcium responses we observed in both the control and the Δ*αβγ* neurons were completely abolished by the application of tetrodotoxin (TTX) or 2,3-Dioxo-6-nitro-1,2,3,4-tetrahydrobenzo[f]quinoxaline-7-sulfonamide (NBQX) and 2-amino-5-phosphonovaleric acid (APV; Figures 4C,D). In contrast, the synchronized response of the Δ*αβγ* neurons was not inhibited by the gap junction blockers meclofenamic acid (MFA) or 18β-Glycyrrhetinic acid (β-GA; Figures 4E,F), suggesting that the synchronized response of the Δ*αβγ* neurons was not caused by gap junctions, but rather by synapse function.

**Figure 5:**
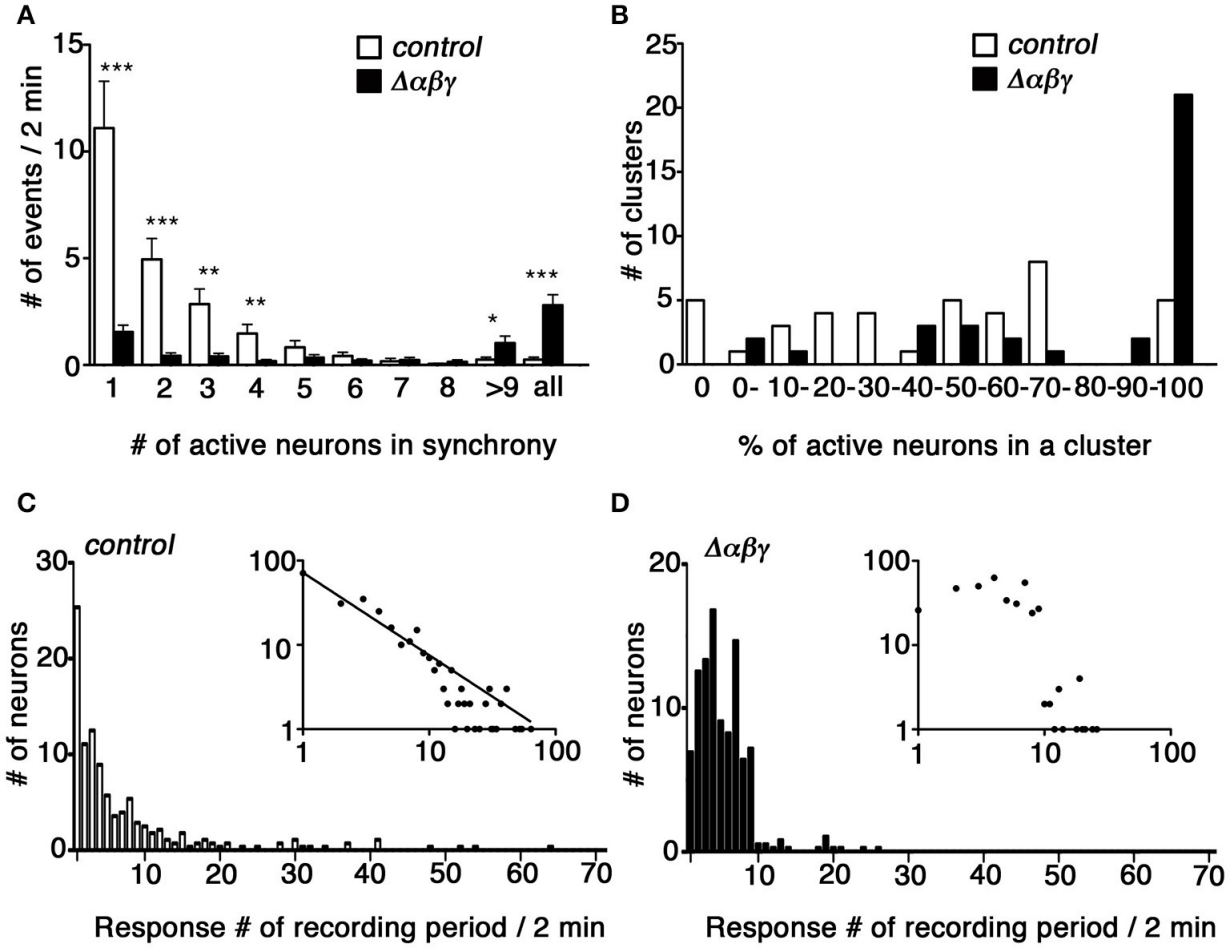
**Synchronous activity was higher in cultured Δ***αβγ*** hippocampal neuronal networks. (A)** Histogram showing the frequency of calcium responses in which the number of neurons indicated in each bin responded in synchrony. Events when all neurons in a cluster responded in synchrony were compiled in the right-most “all” column. Error bars represent SEM, ^***^P < 0.0001, ^**^P < 0.005, ^*^P < 0.05 by Mann-Whitney *U*-test, *n* = 40 control (white) and 35 mutant (black) clusters for each of six independent cultures. **(B)** Histogram showing the percentage of active neurons in each cluster analyzed. The numbers in each bin indicate the percentage of active neurons and correspond to the lower limit range covered by each bin: 0 and 100% were assigned separate bars, and the range between 0 and 100% was divided into 10 bins. *N* = 40 clusters for control (white), and *n* = 35 mutant (black) clusters for six independent cultures each. **(C,D)** Histograms showing the distribution of the number of neurons with the calcium response frequency observed during a 2-min recording period. Insets show the same data plotted in log-log coordinates. A power-law distribution with a slope of −0.98 is indicated in the control graph. *N* = 280 control neurons and 385 mutant neurons for six independent cultures. Data derived from the *Pcdh*α*βγ*^+/+^*:TG*^*taf*7^ and *Pcdh*α*βγ*^*del*/+^*:TG*^*taf*7^ genotypes were used as controls.

To analyze the degree of synchronicity more closely, we imaged medium-sized clusters (9–25 cells) and generated histograms showing the distribution of response frequency with respect to synchrony size (i.e., the number of neurons responding in synchrony; Figure 5A). In control clusters, the most frequent events were solo activities, and the number of events decreased as the size of the cluster of simultaneously active neurons increased to a completely synchronized state. In contrast, the most frequent events in Δ*αβγ* networks were the completely synchronous activities of all of the neurons in a cluster (Figure 5A, right-most column). The control and Δ*αβγ* cultures also differed in the percentage of active neurons in a cluster (Figure 5B). Control clusters showed a wide-ranging and roughly even distribution of active neurons during a 2-min imaging period (no active neurons in five clusters, 1–3 active neurons in five clusters, or complete synchronization of all neurons in five clusters). In contrast, the Δ*αβγ* clusters had a higher percentage of active neurons, with 60% of the imaged clusters showing complete synchronization of all the neurons in the cluster (Figure 5B).

Finally, we analyzed the calcium response frequency for each neuron. The histograms in Figures 5C,D show the distribution of neuron counts relative to the response frequency. In control neurons, the least active neurons (only a single response during a recording period) accounted for the majority of the neurons, and the number of neurons decreased as the response frequency increased. The distribution had a heavy right tail, and we occasionally encountered neurons with high-frequency responses ranging from 30 to 64 responses per 2-min recording period (Figure 5C). In contrast, the distribution in Δ*αβγ* neurons was bell-shaped, with 1–10 responses per recording period, and we never encountered neurons with response frequencies higher than 30 (Figure 5D). This difference was more evident when the histogram data were plotted on a log-log scale (Figures 5C,D, insets). In the case of control neurons, the distribution was well-fitted to a power-law distribution with a slope of −0.981; this relationship was not present in the Δ*αβγ* neuron cultures.

These results revealed a difference in the neuronal activity propagation in control vs. Δ*αβγ* networks; in control cultures, the neuronal activity usually propagated within confined small groups of neurons, while in Δ*αβγ* cultures, the neuronal activity easily propagated to the entire network. This observation suggests that the clustered Pcdh isoforms are required to build neural circuits consisting of neuronal assemblies of various sizes.

### *Trans*-homophilic interactions of γA3 isoforms in *Δ*αβγ** neurons

The results obtained so far suggest that Pcdh isoforms are required for appropriate wiring of neural circuits, and that *trans*-homophilic interaction of identical Pcdh isoforms underlies this process. However, the specificity of *trans*-interactions of Pcdh isoforms has not been tested in primary neurons yet. Using the K562 cell line, which lacks endogenous clustered Pcdh isoforms expression, Schreiner and Weiner demonstrated that Pcdhγ-mediated *trans*-homophilic cell-cell interactions occur between *cis*-heteromeric multimers (Schreiner and Weiner, 2010; Thu et al., 2014). Based on this finding, using live Δ*αβγ* neurons, we sought to capture the formation of homomeric complexes consisting of a single Pcdh isoform within individual neurons and the expected *trans*-homophilic neuron-neuron interactions.

**Figure 6:**
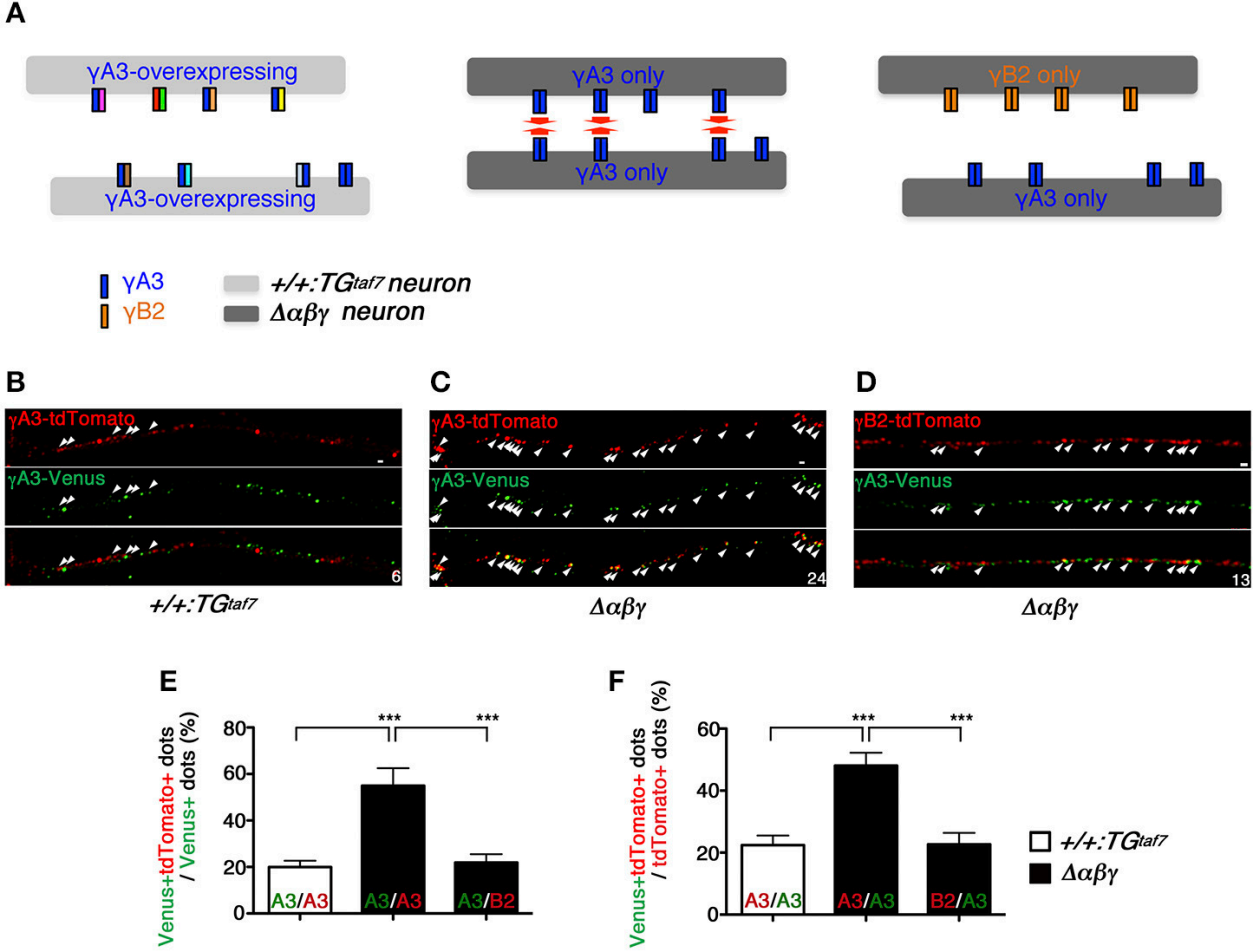
**Pcdh single-isoform overexpression in dissociated neurons revealed exclusively homophilic neuron–neuron interactions in trans. (A)** Schematic diagram of the highly frequent *trans*-homophilic interactions of the γA3 isoforms between Δ*αβγ* neurons. To simplify the demonstration, each isoform complex is shown as a dimer. **(B,C,E,F)** E16.5 hippocampal neurons were nucleofected with γA3-tdTomato or γA3-Venus expression vectors and co-cultured for 11 days. Significantly more tdTomato^+^ and Venus^+^ dots were colocalized in the hippocampal neurons of Δ*αβγ* mutants, compared to those of the +*/*+*:TG*^*taf*7^ mice. The small numbers at the corners indicate the number of colocalized yellow dots (arrows). **(D)** A similar experiment with different Pcdh isoforms (γB2-tdTomato and γA3-Venus) in Δ*αβγ* mutants showed that the homophilic interaction was an isoform-specific feature that was only detectable in the Δ*αβγ* neurons. Immunostaining revealed microscope fields in which tdTomato^+^ and Venus^+^ neurites ran parallel within a distance of <0.5 μm of each other. Data were analyzed by one-way ANOVA and *post-hoc* Tukey tests. The numbers of neurite pairs analyzed were as follows: γA3-γA3, 11 control (+*/*+*:TG*^*taf*7^) and 16 Δ*αβγ*; γA3-γB2, 20 in mutant Δ*αβγ*. Error bars represent SEM; ^***^P < 0.001. Bars: 0.5 μm.

We expected that the exogenous single isoform-mediated *trans*-homophilic interactions occurred only in Δ*αβγ* neurons lacking all of the endogenous clustered Pcdh isoforms. On the other hand, the endogenous clustered Pcdh isoforms that are stochastically and differentially expressed in control neurons might interfere with the isoform-specific *trans*-homophilic interactions of the single isoforms between neurons (Figure 6A).

For the analysis, we selected Δ*αβγ* hippocampal neurons because WT hippocampal neurons express clustered Pcdh isoforms at high levels (Katori et al., 2009) and also because they developed normally until DIV 21 without neuronal death, extending axons and dendrites and forming both excitatory and inhibitory synapses that differed little in appearance or number from those observed in the control cultures (Supplementary Figure 4). In +*/*+*:TG*^*taf*7^ and Δ*αβγ* hippocampal neurons, we first overexpressed the γA3 protein, which was previously shown to have cell-adhesion activity in K562 cells (Schreiner and Weiner, 2010; Thu et al., 2014), and analyzed its homophilic interactions. The overexpressed γA3 protein was tagged with Venus or tdTomato, large numbers of copies were independently transfected into distinct neurons, and the neurons were then co-cultured for 11 days in the same dish. Neurons overexpressing the γA3 protein in +*/*+*:TG*^*taf*7^ culture or Δ*αβγ* culture similarly extended their processes, and there were no differences in the intensity or distribution pattern of fluorescence between both genotypes at DIV 11 (Supplementary Figure 5). In this study, overexpressed γA3-Venus or γA3-tdTomato was localized in neurites as puncta. These distribution patterns were similar to that of the overexpressed γA3-GFP on the neurites in the previous paper (Fernandez-Monreal et al., 2009). Therefore, in this study we focused on the neurite pairs in close contact running side-by-side.

To assess the *trans*-homophilic interactions between γA3-Venus and γA3-tdTomato originating from different neurons, we counted the overlapping Venus and tdTomato signals in neurites running side-by-side within a distance of <0.5 μm of each other (Figures 6B,C). The γA3-Venus and γA3-tdTomato signals overlapped frequently in Δ*αβγ* neurons, but rarely in control neurons (Figures 6E,F). Similarly, these results were also confirmed in medullary neurons, which have neuronal death phenotype (Supplementary Figure 6). Furthermore, the signals from neurons expressing the different isoform γB2-tdTomato rarely overlapped with γA3-Venus, even in Δ*αβγ* neurons (Figures 6D–F). We observed parallel-running neurite pairs with high frequency in the exogenous γA3 expressed Δ*αβγ* neurons, while such neurite pairs were rare in the exogenous γA3 expressed control neurons. These results suggested that each of endogenous clustered Pcdh isoforms in neurons acted as a “poison partner” to disrupt the isoform-specific *trans*-homophilic adhesion between the exogenously-introduced γA3 isoforms (Figure 6A).

## Discussion

In this study, we found that the apoptosis of Δ*αβγ* neurons depends on the brain area. We revealed for the first time the characteristics of malfunctioning Δ*αβγ* neural circuits, such as the CPG circuit in the spinal cord, and the cultured hippocampal neuron network. Lastly, using complete-null clustered Pcdh neurons, we showed that the endogenous clustered Pcdh isoforms in neurons interfere with the isoform-specific *trans*-homophilic interactions of clustered Pcdh proteins.

### Clustered-Pcdh-isoforms-mediated *Trans*-homophilic-interactions between neurons

The specificity of *trans*-homophilic cell-cell interactions of *cis*-heteromeric complexes of Pcdh isoforms has been systematically studied in K562 cells. However, neurons also use other recognition systems, such as the ephrin-Eph or the neurexin-neuroligin system. Additionally, a recent study described that Pcdhγ isoforms inhibit synaptogenesis by blocking interactions between neuroligin-1 and neurexin1β (Molumby et al., 2017). Therefore, we tried to clarify whether the “specificity rule” established in cell lines was applicable to neuron-neuron recognition. In our study, by expressing an exogenous Pcdh single isoform in complete-null clustered *Pcdh* mutant neurons, we captured the clustered Pcdh isoforms-mediated *trans*-homophilic interactions in living neurons for the first time. As expected, exogenous γA3 isoforms on two contacting Δ*αβγ* neurons were highly colocalized, confirming the *trans*-homophilic interaction. In contrast, the labeled isoforms were rarely colocalized between control neurons. This could be explained as follows. In the controls, the exogenous γA3 isoform is thought to form *cis*-heteromeric complexes with a diverse set of endogenous clustered Pcdh isoforms, and the resultant variety of *cis*–complexes reduces the probability of an encounter between two identical *cis*-multimers on different neurons. Thus, our results, which are consistent with the data in K562 cells, suggest that a single mismatched clustered Pcdh isoform interferes with the combinatorial homophilic interactions between cells (Schreiner and Weiner, 2010; Yagi, 2012, 2013; Thu et al., 2014). These findings imply that the clustered Pcdh isoforms regulate the specificity of neuronal contacts, and hence the network wiring between different neurons, including dendrite-axon, dendrite-dendrite, and axon-axon contacts. However, we note that there are many differences in the molecular properties of individual 58 clustered Pcdh isoforms; cell surface delivery, intrinsic *trans*-binding affinities, separate roles for neuronal survival or neuronal wiring (Chen et al., 2012; Thu et al., 2014). Moreover, differences also exist in γA and γB subfamilies; Pcdh ectodomains from γB isoforms form *cis*-multimers, whereas γA isoforms do not (Goodman et al., 2016). Therefore, it will be important to carry out a comprehensive analysis for studying the recognition specificity of all of the Pcdh isoforms on neuron-neuron interactions.

### Disorganized output firing pattern of a spinal neuronal network

Since the major anatomical defect in Δ*αβγ* mice was the massive neuronal death in the brainstem and the spinal cord, neuronal degeneration in the respiratory system or in the CPG for locomotion was likely to be the cause of the neonatal lethality of the pups. However, considering the role of clustered Pcdh isoforms in cell-cell recognition, the connection specificity between neurons might also have been affected. Using Δ*αβγ:Bax*^−/−^ double mutants to block the neuronal apoptosis, we first clarified the nature of the clustered Pcdh-deficient neural circuits. In the double mutants, although neuronal degeneration was rescued, the reduced synaptic density was not restored, suggesting a defect in neuronal connectivity. Most importantly, the motor neuron output pattern in the mutant spinal CPG circuits was disorganized, indicating a failure of the functional neural circuits. Although the bath application of NMDA and 5-HT increased the motor neuron activity in the mutants as in WT, the mutant circuits failed to produce the rhythmic left-right alteration seen in the WT circuits. As inhibitory connection plays a role in this alteration, blocking the inhibitory synaptic transmission in WT changed the pattern to a left-right synchronous rhythm. Similarly, the mutants also produced left-right synchronous rhythmic activity upon the blocking of inhibitory synaptic transmission, suggesting that the functional inhibitory synapses were present but wrongly connected. In summary, even though the clustered Pcdh isoforms are dispensable for part of the neural network wiring, they are indispensable for functional network wiring.

### Abnormal synchronized activity in cultured *Δ*αβγ** hippocampal neurons

The network failure of the spinal CPGs observed in the Δ*αβγ:Bax*^−/−^ double-mutant mice suggested that abnormal wiring underlies the malfunction of the mutant circuits. To further examine the substantial change in neural network connectivity, we performed multi-neuron calcium imaging of cultured hippocampal neurons. We used a 100-ms imaging interval to observe a synchronized group of neurons engaged in the same firing episode, which may represent a “cell assembly” sharing information flow. In control networks, the most frequent response was a solo response or a synchronized response of 2–3 neurons. The proportion of active neurons varied among clusters, but usually a subgroup of neurons in a cluster responded. This suggests that the WT network can generate a variety of “cell assemblies” of various group sizes. In contrast, the most frequent response in Δ*αβγ* networks was the complete synchronization of all hippocampal neurons within a cluster. More neurons were actively engaged; in fact, in most Δ*αβγ* clusters, more than 90% of the neurons were active within the imaging period. The higher incidence of synchronization suggests that the Δ*αβγ* network failed to generate the normal repertoire of a “cell assembly” and also failed to prevent the spreading of activity in the entire network. Considering the potential role of Pcdh isoforms in determining the wiring specificity, the Δ*αβγ* neurons showed a network topology greatly changed from that of the WT. It has been shown that the WT neural networks assume a scale-free and small-world topology (Bonifazi et al., 2009; Downes et al., 2012). Our simulation study showed that the stochastic expression of Pcdh isoforms can generate a small-world topology in the network (Kitsukawa and Yagi, 2015). In the Δ*αβγ* network, we speculated that the scale-free and small-world topology was broken by the loss of synaptic partner restriction due to the clustered Pcdh isoforms. In fact, analyzing the distribution of response frequency of each neuron in our culture using a log-log scale revealed that the distribution in control neurons was well-fitted to a power-law distribution, suggesting that the control network might be organized with a scale-free topology, while this distribution was lost in the Δ*αβγ* network. Given the lack of synaptic partner selection in the absence of the clustered Pcdh isoforms, and the observed tendency of the network to synchronize, it seems likely that Δ*αβγ* networks are organized with a random network topology. This idea is in accordance with the results of the network simulation study by Massobrio et al. (2015), which showed that the scale-free network can generate variable sizes of active neuron groups, while the random topology network is not suitable for generating this variety.

### Cause of the massive neuronal death

The neonatal lethality of Δ*αβγ* mutant pups (Hasegawa et al., 2016) is probably due to both the malfunction of neural circuits and the massive neuronal death in the brainstem including the respiratory center. Regarding the cause of the neuronal death in Δ*αβγ* mice, there are at least three plausible explanations. The first possibility is a direct effect of clustered Pcdh signaling, in which the clustered Pcdh proteins transmit a survival signal into the cell.

However, neuronal loss in the *Pcdh*γ-conditional mutants was also previously shown to depend on the surrounding neural network. It was shown that, for at least one type of spinal interneuron, survival did not depend only on whether Pcdhγ isoforms were expressed by that neuron, but also on whether surrounding cells expressed Pcdhγ isoforms. A *Pcdh*γ conditional mutant neuron could survive if surrounded by WT neurons, while a WT neuron surrounded by mutant neurons could undergo apoptosis (Prasad et al., 2008). Considering the possible dependency of apoptosis on the surrounding network and the necessity to make a given number of synapses for neuronal survival, the second possibility is that the Δ*αβγ* neurons failed to form a sufficient number of synapses for their survival. Consistent with this possibility, in the Δ*αβγ* mice, synapse loss was only observed in the death-phenotype neurons (e.g., spinal or reticular neurons) prior to death, but not in the survival-phenotype neurons (e.g., hippocampal or cortical neurons), supporting the concept that neurons with a given number of synaptic inputs will survive.

The last possibility is that the hyper-excitation due to the abnormal synchronized activity in Δ*αβγ* networks may have caused excitotoxicity, which triggered the apoptotic pathway. It has been shown that during network formation, neurons show rhythmic activity that spreads throughout the entire developing network (Feller, 1999; Ben-Ari, 2001; Blankenship and Feller, 2010; Momose-Sato and Sato, 2013). In the brainstem (e.g., the respiratory CPG) and the spinal cord (e.g., the locomotor CPG), the rhythmic activity of the CPGs occurs at a developmental stage similar to that when the massive apoptosis occurred in Δ*αβγ* mice (Branchereau et al., 2000; Nishimaru and Kudo, 2000; Pagliardini et al., 2008). In the spinal cord, the neuronal death was more severe in the ventral than the dorsal interneurons. Considering that heavier input converges in the ventral side close to the output of the circuit, hyper-excitation is another likely cause of the massive neuronal death in Δ*αβγ* mice.

### Survival or death

It is intriguing that the neuronal death was not strictly correlated with whether the neuron normally expressed clustered Pcdh isoforms. For example, spinal motor neurons, cortical neurons, and hippocampal neurons all express clustered Pcdh proteins in WT mice, but the loss of clustered Pcdh proteins did not cause apoptosis in these neuronal types. Similar results have been reported in *Pcdh*γ KO mutants (Wang et al., 2002; Su et al., 2010; Garrett et al., 2012). In contrast, the survival of spinal interneurons, or medullary neurons, is heavily dependent on the presence of clustered Pcdh isoforms. Considering the possible effect of network topology or input synchrony, the evolutionarily old architecture such as the spinal cord and the brainstem may have a different network topology, or a weaker tolerance to strong excitation, compared to the hippocampus and cortex, which evolved later and exhibit a more sophisticated laminar structure. In fact, the brain region that exhibited the most severe apoptosis was the reticular formation in the brainstem. The structure of the reticular formation is not anatomically well-defined, because it includes neurons located in diverse parts of the brainstem, which are heavily interconnected for the integration of information. Therefore, the reticular formation may adopt a network topology that is favorable for input synchrony in WT, and in the Δ*αβγ*, this network may become susceptible and intolerant to heavy input synchrony.

## Conclusions

Our present study of mice lacking all of the clustered Pcdh isoforms demonstrated the significant role of these molecules in building functional neural circuits in the brain. Our results support and extend our recent finding that clustered Pcdh isoforms in neurons regulate the formation and stabilization of connections to establish lineage-specific connection reciprocity (Tarusawa et al., 2016). In addition, the Δ*αβγ* mutant enabled us to manipulate the combinatorial expression of clustered Pcdh isoforms at the individual neuron level. These experiments lay the groundwork for studies addressing novel questions about the relationship between neural network topology and neural activity, as well as the molecular mechanisms by which synaptic connections are built through the *trans*-homophilic interactions of clustered Pcdh isoforms.

## Author contributions

SH, TY, and HKo designed the research and wrote the manuscript. SH and MK performed the immunohistochemistry and analyzed the data. SH and HKa performed the dissociated cultures. HKo performed the calcium-imaging analysis. HN performed the electrophysiological analysis. TH performed the expression vector constructions for γA3 and γB2 isoforms. ET and YY provided basic data for the Pcdh-chimeric mice. MS, MH, and TH contributed to generating the mutant mice.

### Conflict of interest statement

The authors declare that the research was conducted in the absence of any commercial or financial relationships that could be construed as a potential conflict of interest.
