# Supplementary material for: Clustered Protocadherins Are Required for Building Functional Neural Circuits
Source: Front Mol Neurosci. 2017 Apr 24;10:114. doi: 10.3389/fnmol.2017.00114 (PMC5401904; doi:10.3389/fnmol.2017.00114)
Supplement: Supplementary file 6 [file Image1.PDF]

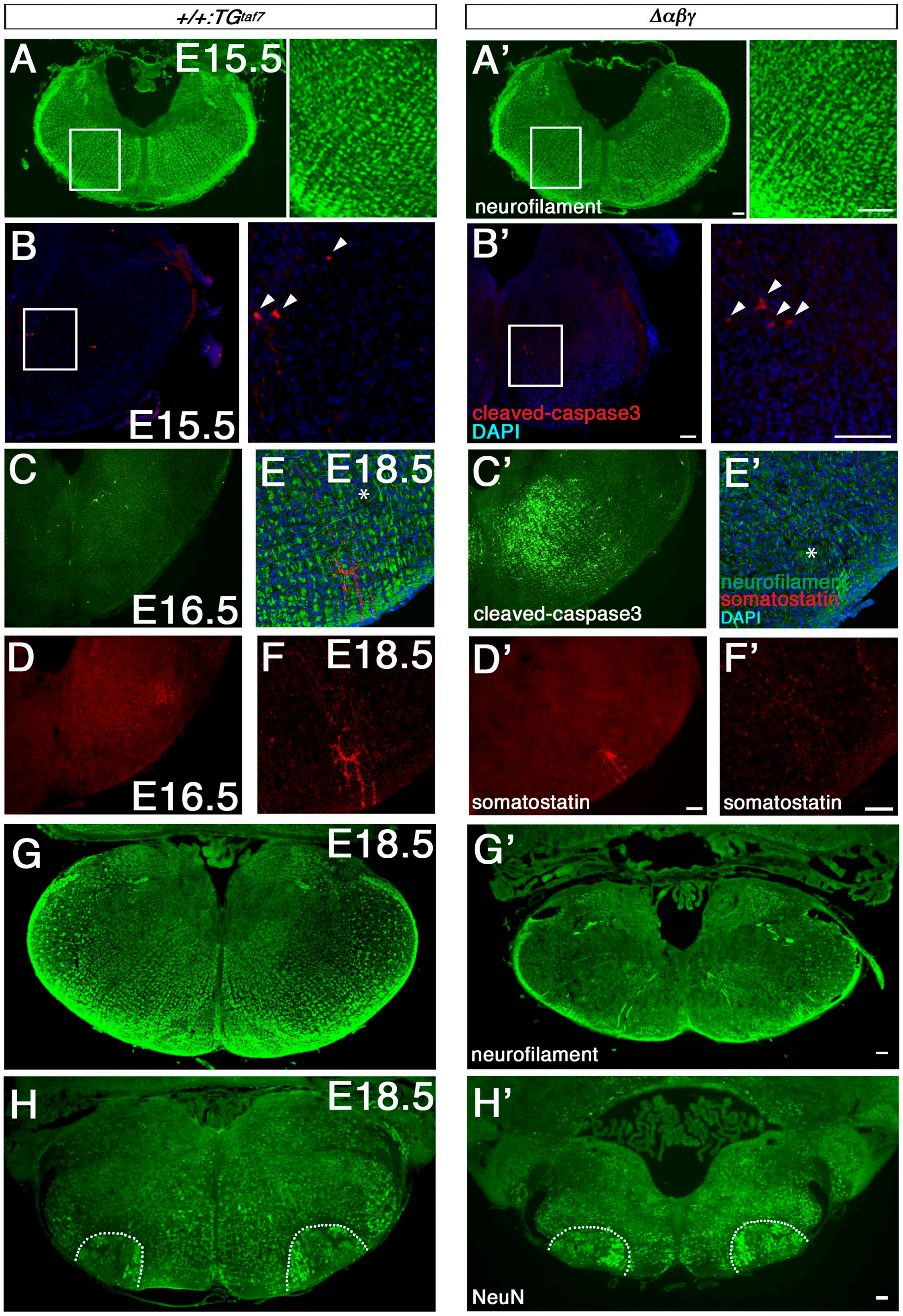

Supplementary Figure 1. Reticular neurons die between E16.5 and E18.5

(A-B') Neurofilament (A', green) and cleaved caspase-3 (B', red) staining in the E15.5 medulla showed no significant differences between the control mice and the *Δαβγ* mutants. Dying cells are indicated by arrowheads (B). (C–C') At E16.5, cleaved caspase-3^{+} signals were strongly detected in the *Δαβγ* mutants. (D–D') Somatostatin staining of the pre-Bötzinger complex (preBötC). (E–F') At E18.5, the net-like pattern of axonal fasciculations was disorganized (E', green), and the somatostatin^{+} interneurons that organize the preBötC were completely absent from the *Δαβγ* mutants (F'). *Ambiguous nuclei present just above the preBötC. (G–G') The medullary size was markedly reduced in the *Δαβγ* mutants. (H–H') NeuN staining of the pons confirmed that the size of the facial nuclei (area encircled by the dotted line) was not altered in the mutants, although massive cell death of interneurons had occurred. Bars: 100 μm.
