# Supplementary material for: Clustered Protocadherins Are Required for Building Functional Neural Circuits
Source: Front Mol Neurosci. 2017 Apr 24;10:114. doi: 10.3389/fnmol.2017.00114 (PMC5401904; doi:10.3389/fnmol.2017.00114)
Supplement: Supplementary file 7 [file Image2.PDF]

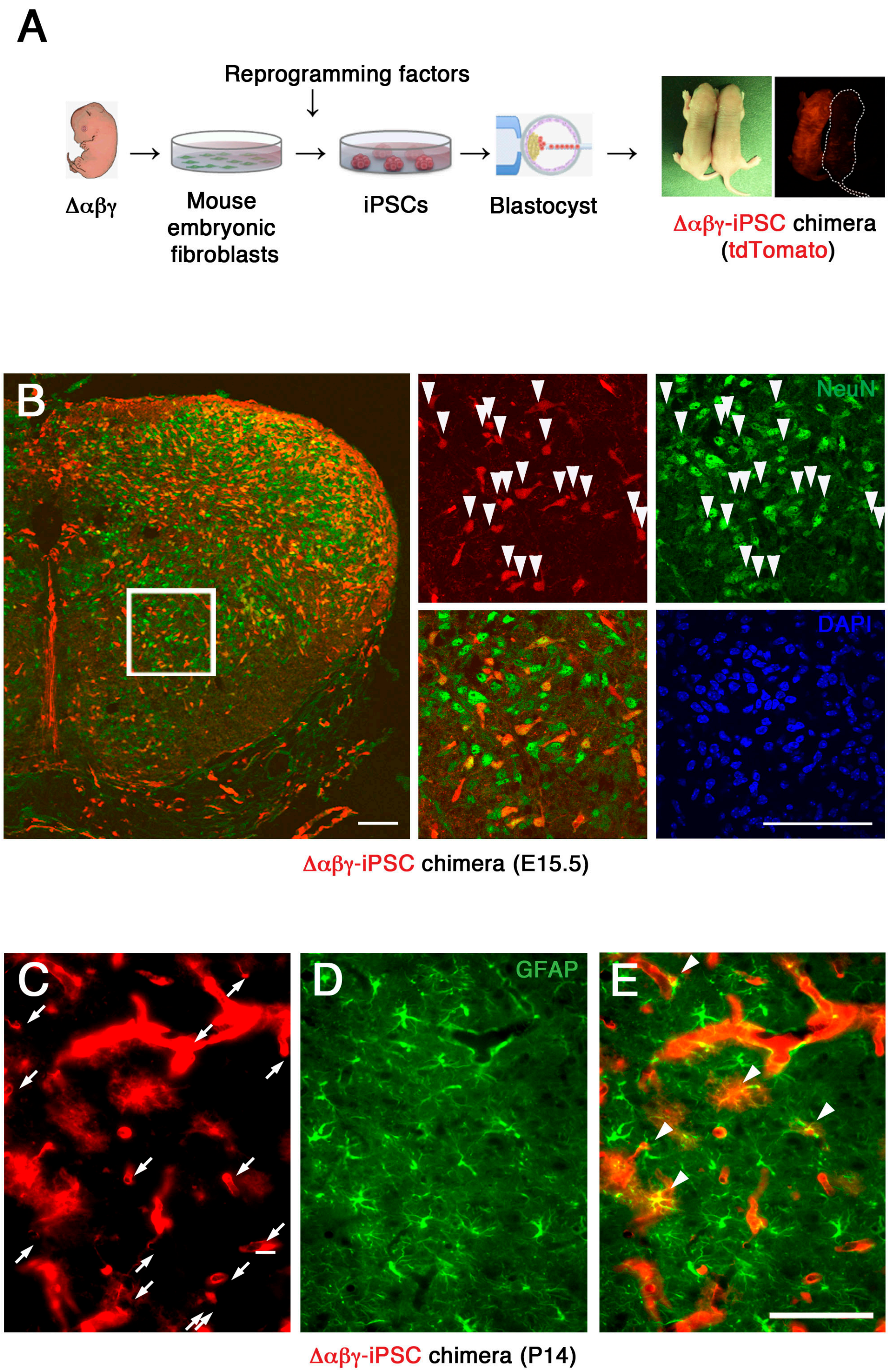

Supplementary Figure 2. ***Δαβγ*** **reticular neurons do not survive even when intermingled with WT neurons**

(A) Generation of *Δαβγ*-iPSC chimeric mice, and two representative P1 *Δαβγ*-iPSC chimeric mice showing different levels of chimerism. (B) Representative image of a medullary section from an E15.5 *Δαβγ*-iPSC chimeric mouse. The magnified view shows the wide distribution of *Δαβγ*-iPSC chimeric neuronal populations. Arrowheads indicate the double positive ($tdTomato^{+}NeuN^{+}$) medullary neurons. (C–E) GFAP staining of the medulla showed increased astrogliosis associated with neurodegeneration and some $tdTomato^{+}$ signals colocalizing with $GFAP^{+}$ signals (arrowheads in E). Many $tdTomato^{+}$ signals were also found in the blood vessels (arrows in C). The *TAF7* transgene was included in the *Δαβγ*-iPSC chimeras, but not in the WT-iPSC chimeras. Bars: 100 µm.
