# Supplementary material for: Clustered Protocadherins Are Required for Building Functional Neural Circuits
Source: Front Mol Neurosci. 2017 Apr 24;10:114. doi: 10.3389/fnmol.2017.00114 (PMC5401904; doi:10.3389/fnmol.2017.00114)
Supplement: Supplementary file 8 [file Image3.PDF]

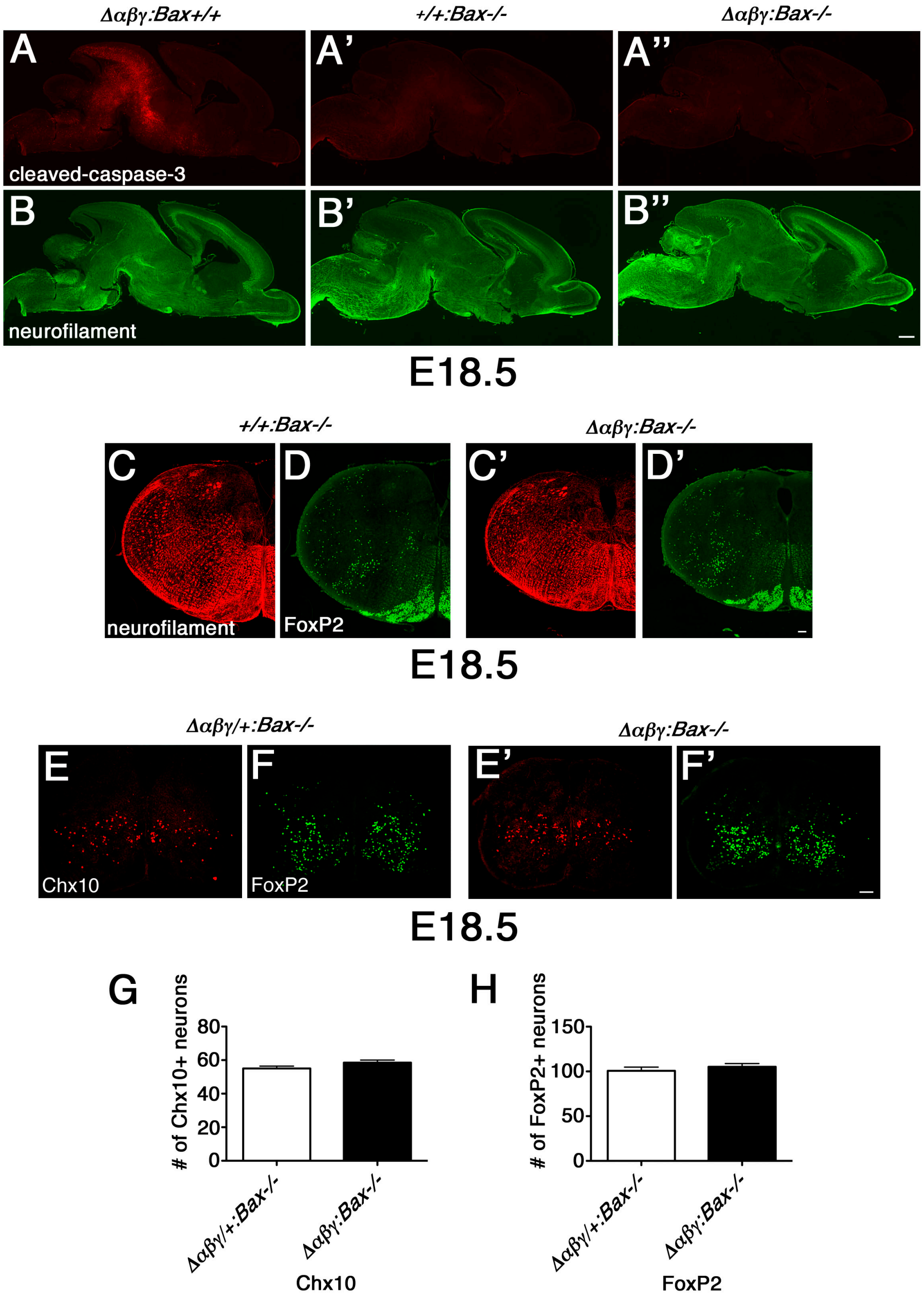

Supplementary Figure 3. Genetic blockage of apoptosis in the *Δαβγ:Bax*$^{-/-}$ double mutants

(A–A'') Cleaved caspase-3 (red) and (B-B'') neurofilament (green) staining of sagittal brain sections of E18.5 *Δαβγ* (A and B), +/+*;TG*^{taf7}*;Bax*$^{-/-}$ (A' and B'), and *Δαβγ;Bax*$^{-/-}$ embryos (A'' and B''). (C and C') Neurofilament (red) and (D and D') FoxP2 (green) staining of coronal sections of the medulla of E18.5 +/+*;Bax*$^{-/-}$ (C and D) and *Δαβγ;Bax*$^{-/-}$ embryos (C' and D'). (E, F) Representative images of Chx10 and FoxP2 immunolabeled E18.5 spinal cords of *Δαβγ/+;Bax*$^{-/-}$ and *Δαβγ;Bax*$^{-/-}$ mice. (G, H) Quantitative analysis of Chx10^{+} (G) and FoxP2^{+} (H) interneuron subpopulations in the ventral horn of the lumbar spinal cord. Error bars represent SEM. In this figure, *TG*^{taf7} was included in each genotype. Bars: 500 µm in (B''); 100 µm in (D',F').
