# Supplementary material for: Clustered Protocadherins Are Required for Building Functional Neural Circuits
Source: Front Mol Neurosci. 2017 Apr 24;10:114. doi: 10.3389/fnmol.2017.00114 (PMC5401904; doi:10.3389/fnmol.2017.00114)
Supplement: Supplementary file 9 [file Image4.PDF]

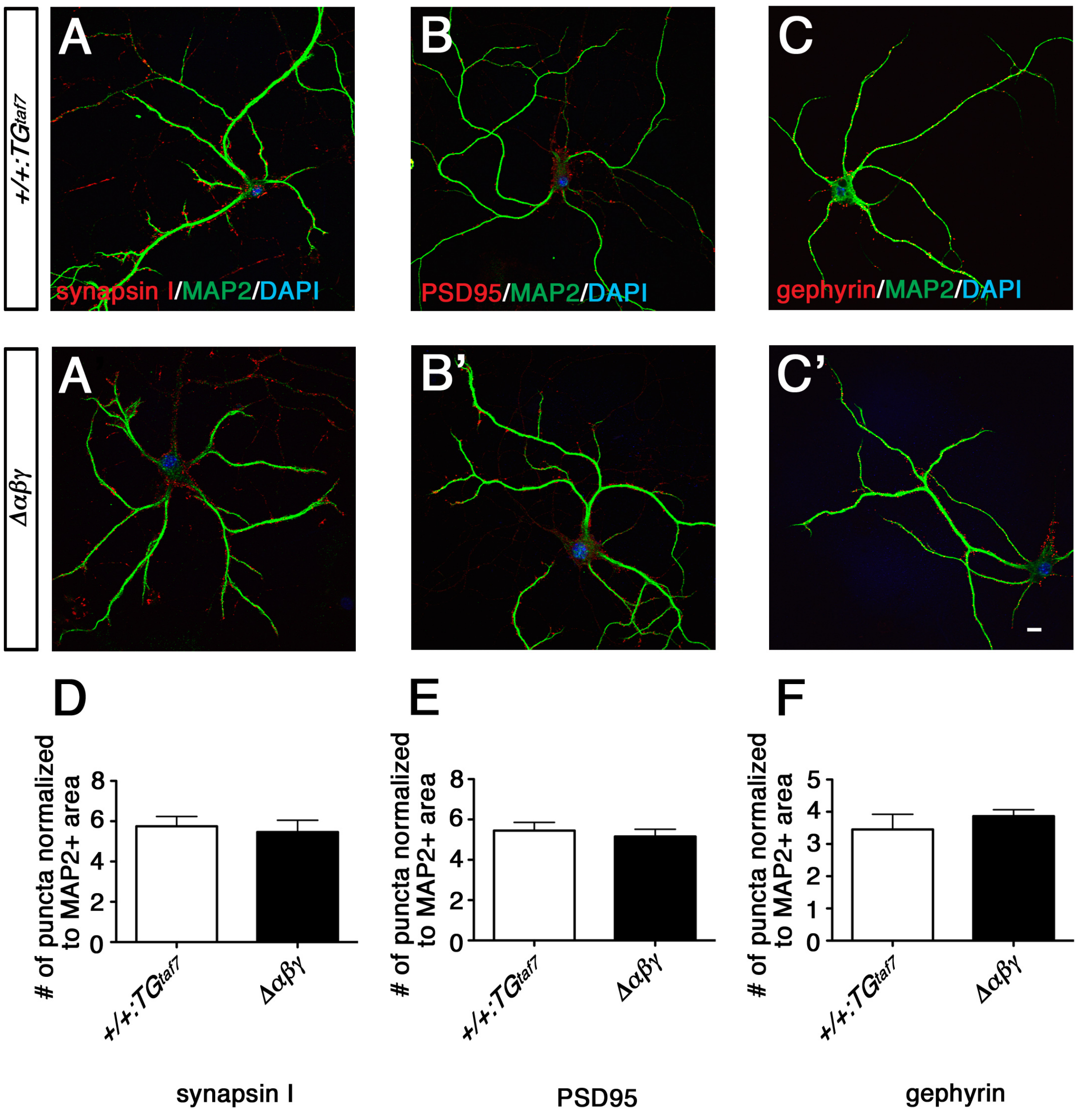

Supplementary Figure 4. Cultured *Δαβγ* hippocampal neurons

(A–C') Representative images of DIV 21 hippocampal neurons stained with synapsin I, PSD95, and gephyrin. Both excitatory (synapsin I in D; PSD95 in E) and inhibitory (synapsin I in D; gephyrin in F) synaptic densities were unaltered in *Δαβγ* neurons; the cell density and $MAP2^+$ dendritic areas were also comparable to the $+/+;TG^{taf7}$ (data not shown). Error bars represent SEM; data were compared by Student's *t* test. We examined 12 $+/+;TG^{taf7}$ and 15 *Δαβγ* neurons in (D), 27 $+/+;TG^{taf7}$ and 29 *Δαβγ* neurons in (E), and 18 $+/+;TG^{taf7}$ and 24 *Δαβγ* neurons in (F). Bar: 10 μm.
