# Supplementary material for: Clustered Protocadherins Are Required for Building Functional Neural Circuits
Source: Front Mol Neurosci. 2017 Apr 24;10:114. doi: 10.3389/fnmol.2017.00114 (PMC5401904; doi:10.3389/fnmol.2017.00114)
Supplement: Supplementary file 10 [file Image5.PDF]

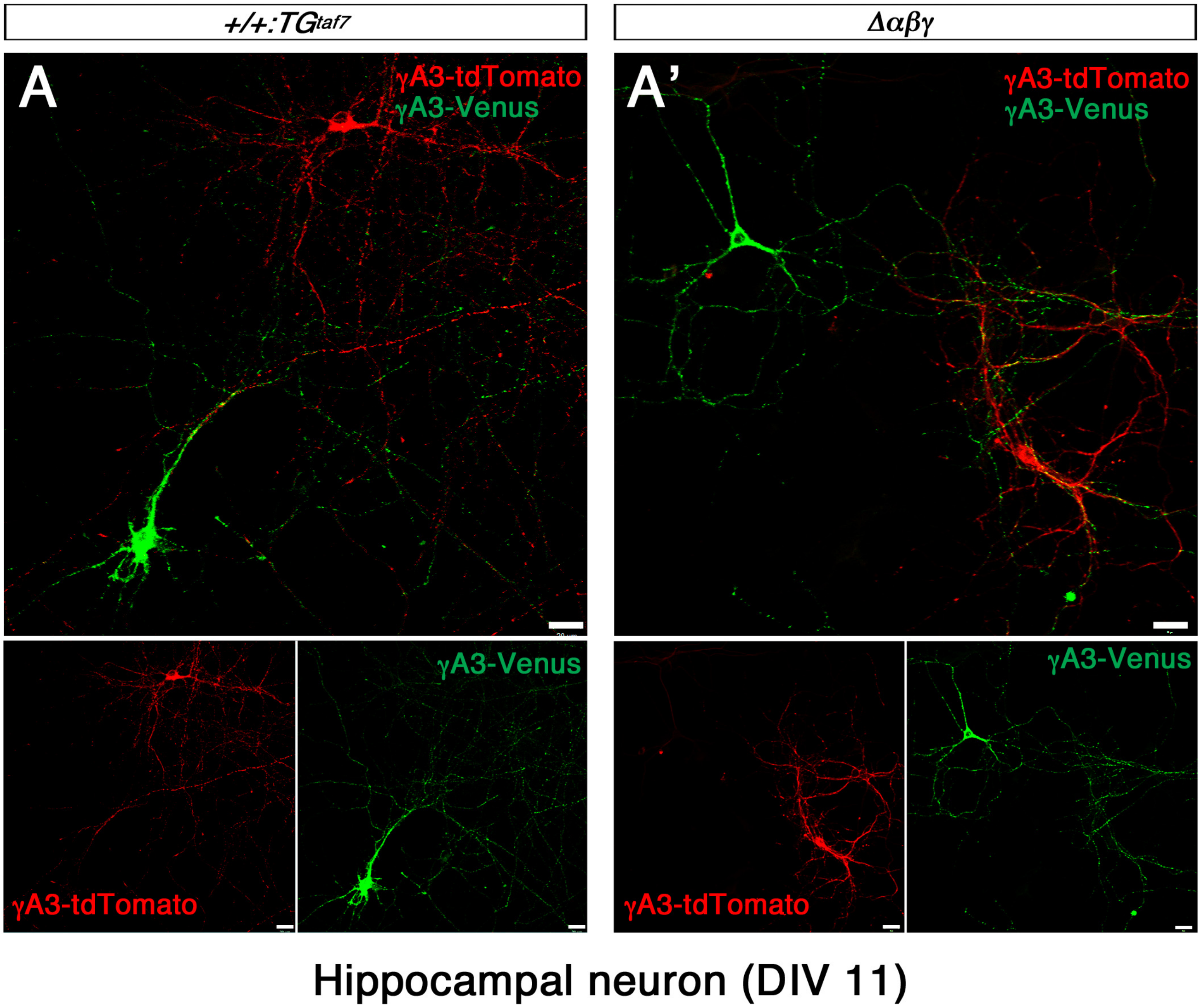

Supplementary Figure 5. Overexpression of γA3 isoforms in *+/+:TG*^{taf7} **or** ***Δαβγ*** **hippocampal neurons**

(A-A') The overexpressed γA3 protein was tagged with Venus or tdTomato, large numbers of copies were independently transfected into distinct neurons, and the neurons were then co-cultured for 11 days in the same dish. Neurons overexpressing the γA3 protein in *+/+:TG*^{taf7} culture (A) or *Δαβγ* culture (A') similarly extended their processes, and there were no differences in the intensity or the distribution pattern of the total amount of the fluorescent signals from the exogenous-expressed γA3 (γA3-Venus or -tdTomato) between both genotypes. At such lower magnifications, we could not show an inset indicating the neurite pairs running side-by-side within a distance of < 0.5 µm of each other. The analysis of the parallel-running neurite pairs was directly quantified using high-resolution images obtained on an ZEISS confocal microscope with an X2.3 digital zoom and X100 oil immersion objective lens. Bars: 25 µm.
