# Supplementary material for: Clustered Protocadherins Are Required for Building Functional Neural Circuits
Source: Front Mol Neurosci. 2017 Apr 24;10:114. doi: 10.3389/fnmol.2017.00114 (PMC5401904; doi:10.3389/fnmol.2017.00114)
Supplement: Supplementary file 11 [file Image6.PDF]

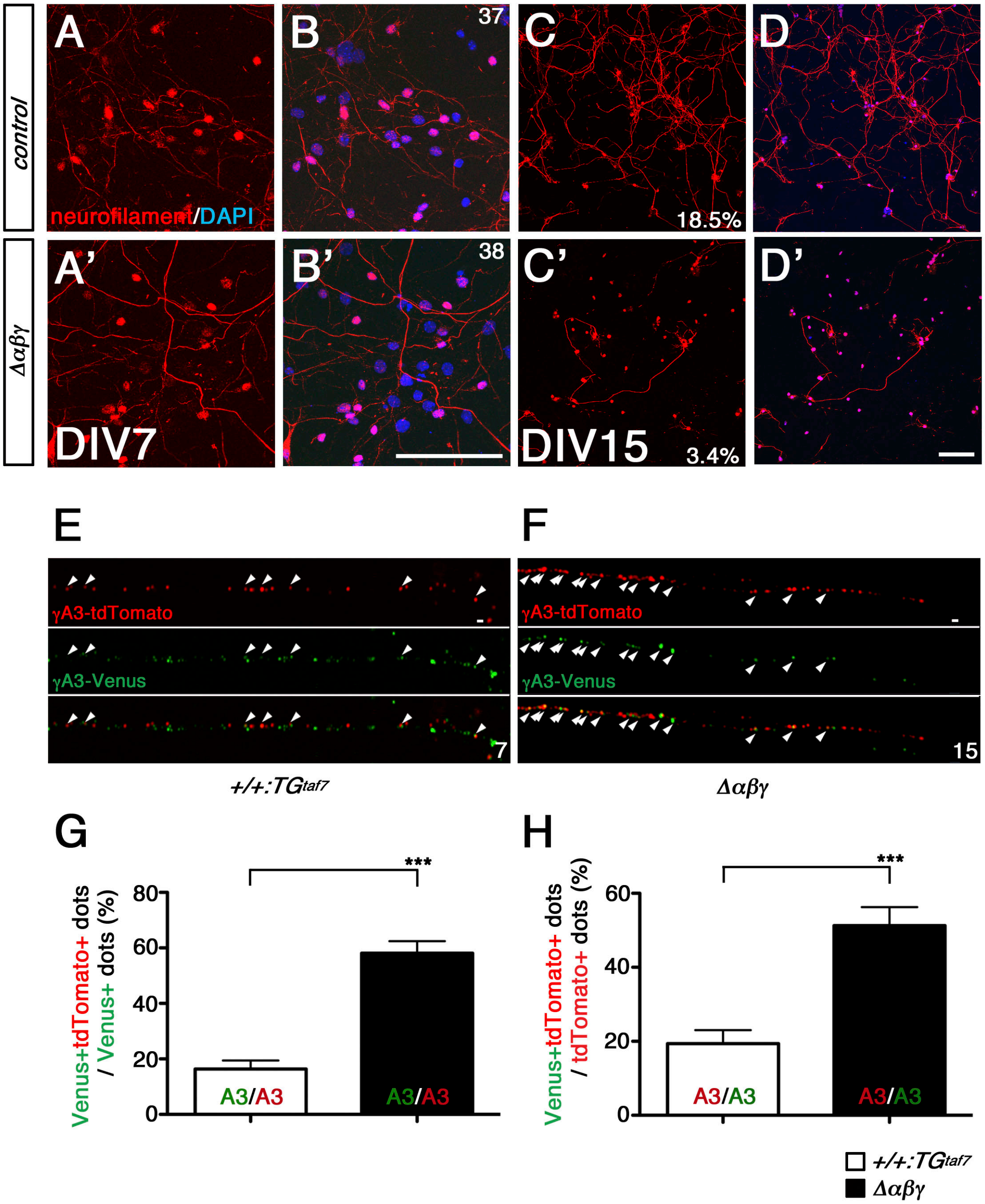

Supplementary Figure 6. Neuronal loss and *trans*-homophilic interactions of γA3 isoforms in cultured *Δαβγ* reticular neurons

(A-B') Cultured reticular neurons at DIV7 were stained with 4',6-diamidino-2-phenylindole (DAPI) and pan-axonal neurofilament (SMI-312). No differences were observed between the control cells (n = 37 cells) and *Δαβγ* (n = 38 cells) cells. (C–D') However, at DIV15, the *Δαβγ* neurons had almost disappeared, accompanied by apoptotic nuclear condensation (D'). (E–H) E12.5 reticular neurons were nucleofected with γA3-tdTomato or γA3-Venus expression vectors and co-cultured for 7 days. *Trans*-homophilic interactions between γA3 isoforms were observed exclusively in *Δαβγ* neurons. Data were analyzed for statistical significance using Student's *t* test. For γA3-γA3, 22 control and 15 mutant neurite pairs were analyzed. Error bars represent SEM; ***$P < 0.001$. The small numbers at the corners indicate the number of colocalized yellow dots (arrows). Immunostaining revealed microscope fields in which tdTomato^{+} and Venus^{+} neurites ran parallel within a distances of < 0.5 µm of each other. Bars: 100 µm in (B', D'); 0.5 µm in (E, F).
